# New Oxindole-Bridged Acceptors for Organic Sensitizers: Substitution and Performance Studies in Dye-Sensitized Solar Cells

**DOI:** 10.3390/molecules25092159

**Published:** 2020-05-05

**Authors:** Yogesh S. Tingare, Chaochin Su, Ming-Tai Shen, Sheng-Han Tsai, Shih-Yu Ho, Wen-Ren Li

**Affiliations:** 1 Institute of Organic and Polymeric Materials/Research and Development Center for Smart Textile Technology, National Taipei University of Technology, Taipei 10608, Taiwan; kieferboy8@hotmail.com (S.-H.T.); stacy760414@hotmail.com (S.-Y.H.); 2 Department of Chemistry, National Central University, Chung-Li 32001, Taiwan; moodplayer@hotmail.com

**Keywords:** oxindoles, halogens, electron injection, dye-sensitized solar cells

## Abstract

New D-*π*-A configured organic sensitizers featuring halogen-substituted oxindole-bridged acceptor units have been synthesized for dye-sensitized solar cells applications. Among fluorine, bromine, and iodine substitution, the cell based on bromine incorporated dye exhibited the highest efficiency. The oxindoles in these sensitizers were found to assist the electron injection through the chelation of their amide carbonyl groups to the TiO_2_ surface. This study provides an alternate approach for future rational dye design to gain excellent DSSC performance.

## 1. Introduction

Energy harvesting directly from the sunlight using the photovoltaic technology is being increasingly recognized as an essential strategy for future global energy production [1]. Finding energy generating devices using inexpensive and environment-friendly materials has become a well-focused academic and industrial research topic. The solar-to-power converting assembly, known as dye-sensitized solar cells (DSSCs), introduced by O’Regan and Grätzel has attracted a significant amount of attention [2,3,4,5,6,7,8,9,10,11,12,13,14,15]. The technology has shown a great potential to assist as an efficient, low production cost, and clean energy source. To achieve excellent solar power conversion efficiencies in DSSCs, considerable attention has been focused on designing and synthesizing various photosensitizers [10,11,16,17,18,19,20]. For the past decades, the sensitizers like ruthenium (II) polypyridyl complexes [21,22,23,24,25,26,27] and zinc-based porphyrin dyes [28,29,30,31,32,33] have dominated DSSCs as efficient sensitizers with their outstanding power conversion efficiencies. But the cost, toxicity and limited availability of the metals, as specially the ruthenium, have inspired researcher to pursue alternative sensitizing dyes for DSSC. Parallelly, the ongoing research on perovskite solar cells [34,35] is found to be promising with efficiency exceeding over 20%. But the use of environmentally harmful lead-based materials and instability in these devices have raised some major concerns [36,37]. On the other hand, the metal free organic dyes have kept a considerable interest with their robust availability, flexibility of structural tuning, and high molar extinction coefficients which make them competitive alternatives to the other sensitizers [38,39].

Typically, metal-free organic dyes are configured with donor-π-bridge˗acceptor structures. As per the literature reports, in-depth studies have been directed towards the modification of the donors and the π-bridge moieties of the organic dyes but only a few developments were made in the acceptor groups [10,40,41,42]. The most commonly used acceptors (anchoring groups) are carboxylic acid or cyanoacrylic acid [9]. But the cyanoacrylic acid acceptor was reported to exhibit some disadvantages, such as lower photostabilities due to presence of double bond and dominating redox properties because of strong electronegative nitrile moiety [43]. It was also reported that the nitrile group of the cyanoacrylic acid affected the acidity of the adjacent carboxylic acid which can give a negative influence on the binding to the TiO_2_ surface [44,45]. Several attempts have been made to replace the strong electron-withdrawing cyano group and construct new acceptor systems to achieve high absorptivity and good photostability [46,47,48,49]. The acceptor moiety of the dye shows a significant influence on the spectral response and the device performance. Therefore, there is a need to further explore various acceptors and expand “design principles” to help guide metal-free dye discovery.

The oxindoles are natural indole alkaloids and can be isolated from the plants [50]. Although oxindoles are ubiquitous within the pharmaceutical industry [51,52,53], the potential of oxindoles to act as sensitizing materials [54] has not been investigated by other groups and need to be studied thoroughly. We unprecedentedly visualize oxindoles as new acceptors for the organic sensitizers. It is our speculation that the amide group of the oxindole dye will not only act as an electron acceptor but also can direct the electron injection because of its chelation to the TiO_2_ surface [54]. Furthermore, it is located near the anchoring group which will help to accelerate the electron transfer to the TiO_2_ due to the shorter distance. The non-conjugated carboxylic acid group incorporated in the end of the dye will act as efficient binding group without affect the push–pull capability of the acceptor. Effect of halogens substituted on the donor moiety of the dye was also studied and was found to effectively contribute to the enhancement of the open-circuit voltage and the overall cell efficiency [55]. 

**Figure 1:**
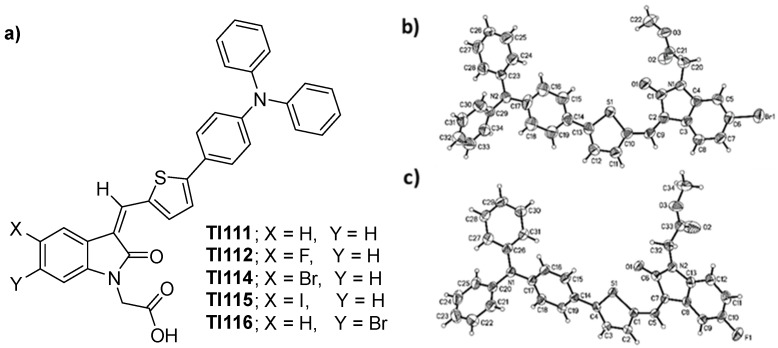
(**a**) Molecular structures of oxindole dyes (**TI111**−**TI116**). (**b**) ORTEP diagram of methyl ester of **TI112**. (**c**) ORTEP diagram of methyl ester of **TI116**.

In this article, we designed and synthesized a series of new oxindole sensitizers (**TI111**–**TI116**, Figure 1a) which exhibited respectable photoelectric conversion efficiencies (*η*). These sensitizers have an excellent electron-donating triphenylamine (TPA) donor and the thiophene in the spacer and are differentiated by various halogen-substituted oxindole acceptors. The electronegative fluorine (F) element is well known for its application in the field of organic light-emitting diodes as well as in the artificial amino acid synthesis [56,57,58,59,60]. The alteration in the Fermi levels of the dye could be achieved by using F substituent in the acceptor of the dye, which usually accounts for an increase in the open-circuit voltage of the dye by reducing the dark current [58]. We incorporated an F atom in the parent compound **TI111**, forming **TI112**, to alter the photo-physical properties of the oxindole dye. The comparative studies of different halogen substituents in the donor part of sensitizers have been evaluated [55], but their effects on the sensitizing properties of dyes when substituted near the acceptors have not been reported. Therefore we further extended our investigation and designed **TI114**, and **TI115** in which bromine (Br), and iodine (I) substituents were incorporated, respectively. Additionally, it is noted that the variation in the position of the substituent with respect to the anchoring group can deliver different cell performances [58]. Thus, we designed **TI114** analogue, **TI116**, to study the effect of position of the substitution on the conversion efficiency of the DSSC.

## 2. Results and Discussion

**Scheme 1:**
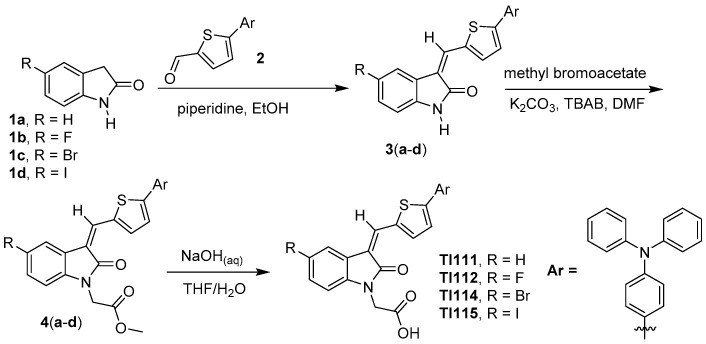
Synthesis of TI111-TI115 sensitizers.

**Scheme 2:**
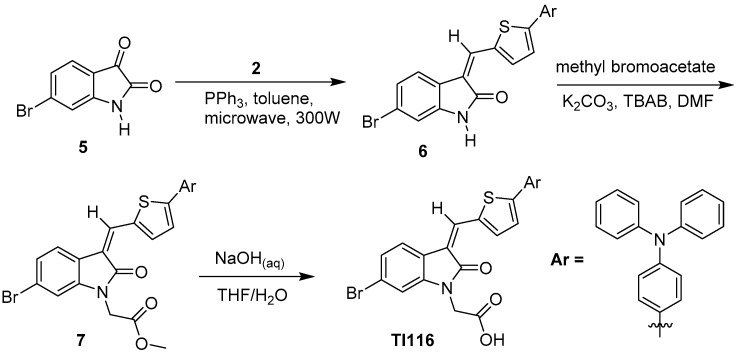
Synthesis of **TI116** sensitizer.

Scheme 1 depicts the syntheses of new sensitizers **TI111**–**TI115** that started with a Knoevenagel condensation reaction using amides **1a**–**d** and 5-[4-(diphenylamino)phenyl]thiophene-2-carbaldehyde (2) [54] to yield condensed compounds **3a**–**d** as single Z-isomers. Oxindoles **3a**–**d** were then alkylated with methyl bromoacetate in the presence of base and tetrabutylammonium bromide (TBAB) to afford esters **4a**–**d**. Finally, sensitizers **TI111**–**TI115** were obtained after basic hydrolysis of their corresponding esters. As shown in Scheme 2, the preparation of the sensitizer **TI116** began with a microwave-assisted Wittig reaction. 6-Bromo-1H-indole-2, 3-dione (**5**), aldehyde **2**, PPh_3_, and toluene were combined in a microwave reaction vessel and irradiated in the Synthos 3000 microwave oven at 300W for 4 h to yield the desired product **6**. Ester **7** was synthesized through alkylation of compound **6** with methyl bromo acetate. Basic hydrolysis of the resulting ester **7** successfully provided the required sensitizer **TI116**. The molecular geometries of **TI112** and **TI116** dyes were confirmed through single-crystal structural analysis of their corresponding esters **4b** and **7**, respectively (Figure 1b,c; Tables S1 and S2 in the Supporting Information). As shown in the X-ray structures, the stereogenic double bonds present in oxindoles are arranged in Z configuration.

**Figure 2:**
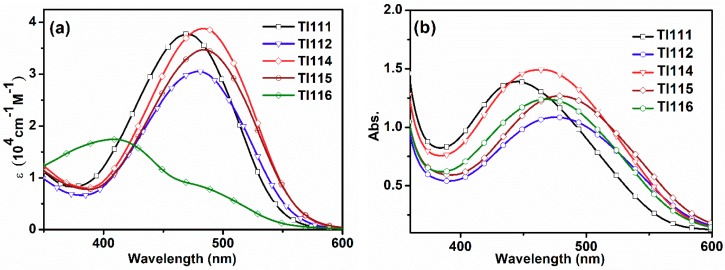
(**a**) Absorption spectra of **TI111**-**TI116** in CH_2_Cl_2_. (**b**) Absorption spectra for transparent TiO_2_ films (thickness: 5 µm) coated with 8.0 × 10^−5^ M solutions of **TI111**–**TI116**.

The optical properties of these dyes were investigated using UV/Vis absorption spectroscopy and shown in Figure 2. The absorption spectrum of the parent dye **TI111** features λ_max_ of 469 nm [molar absorption coefficient (*ε*) =3.77 × 10^4^ M^−1^ cm^−1^] which is attributed to the delocalized π-π* transition (Figure 2a). The halogen substituents played an important role in the absorption properties of the corresponding dyes. The λ_max_ of the dyes **TI112** (480 nm, *ε* =3.05 × 10^4^ M^−1^ cm^−1^) with F substituent was found to red-shift 11 nm. However, decrease in molar extinction coefficient was observed when compared to the non-substituted dye **TI111**. Nevertheless, dye **TI114** (λ_max_ = 483 nm, *ε* = 3.88 × 10^4^ M^−1^ cm^−1^) with Br substituent exhibited the highest λ_max_ and *ε*. Surprisingly, dye **TI115** (λ_max_ = 483 nm, *ε* = 3.47 × 10^4^ M^−1^ cm^−1^) showed lower *ε* in which Br is replaced with I substituent. These results revealed that, in comparison to other halogenated dyes, the Br substitution in the oxindole dye was more effective towards improving the light-harvesting capacity. It is interesting to note that, changing the position of substituent with respect to the anchoring group in **TI116** showed a steep decrease in the absorption intensity, compared to the **TI114** dye which might have resonance interaction of the substituent with the anchoring group. Mostly, organic dyes featured blue-shifts in the absorption bands when attached onto the TiO_2_ surface. Such a phenomenon was attributed to deprotonation of the carboxylic acid or the formation of aggregation of the sensitizers [61,62,63,64,65,66]. The UV/Vis absorption spectra of oxindole dyes anchored onto TiO_2_ films (Figure 2b) were broadened. Compared to the solutions, sensitizers **TI112**–**TI115** on films exhibited stronger adsorption than the dye **TI111**.

**Figure 3:**
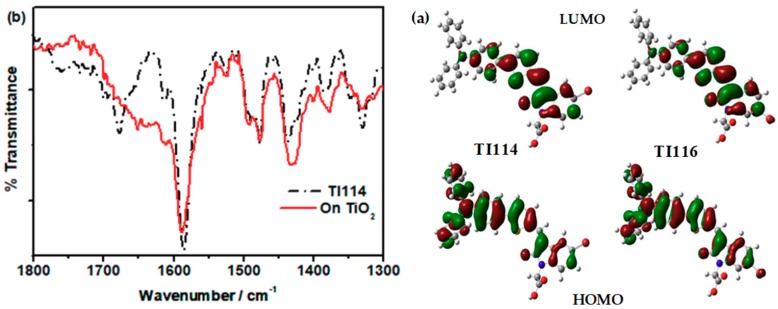
(**a**) Frontier molecular orbital profiles of **TI114** and **TI116** at the B3LYP/6-31g (d,p) level. (**b**) FTIR spectra of the **TI114** dye powder and dye adsorbed on film.

The HOMO and LUMO energy levels of the sensitizer play an important role in affecting the dye-regeneration and electron-transfer processes in DSSCs. Table 1 reveals that the HOMO levels of these dyes are lower than that of I^−^/I^3−^ redox potential, assuring regeneration of the oxidized dyes by the redox couple in the electrolyte. The LUMO levels of the sensitizers **TI111**–**TI116** were adequate for efficient electron injection and the halogen substitutions do not alter the energy levels of these dyes. To gain further insight into the frontier orbital profiles, the locations of the HOMOs and LUMOs of oxindole dyes were calculated using a semiempirical computation method. The molecular orbital calculations showed that the HOMO orbital of each dye is localized to the electron-rich TPA moiety (Figure 3a and Figure S2 in the Supporting Information). Interestingly, in each oxindole dye, the LUMO level is confined to the amide portion of the molecule and does not extend to the non-conjugated anchoring carboxylate moiety. These calculations indicate that each oxindole dye is poised for effective electron injection through the amide group rather than the anchoring carboxylate moiety. To confirm this inference and explore the electron injection mechanism in the oxindoles, FTIR spectra of **TI114** powder and the dye adsorbed on TiO_2_ were measured. The characteristic band for the amide carbonyl group of the oxindole dye **TI114** powder was observed at 1678 cm^−1^ (Figure 3b). When the dye was adsorbed on the TiO_2_ surface, the peak for C=O stretching disappeared which clearly verified the chelation of the amide carbonyl group of the oxindole dye to the TiO_2_ surface. With such chelations, oxindole sensitizers are expected to improve photo-induced electron injection yields. 

**Figure 4:**
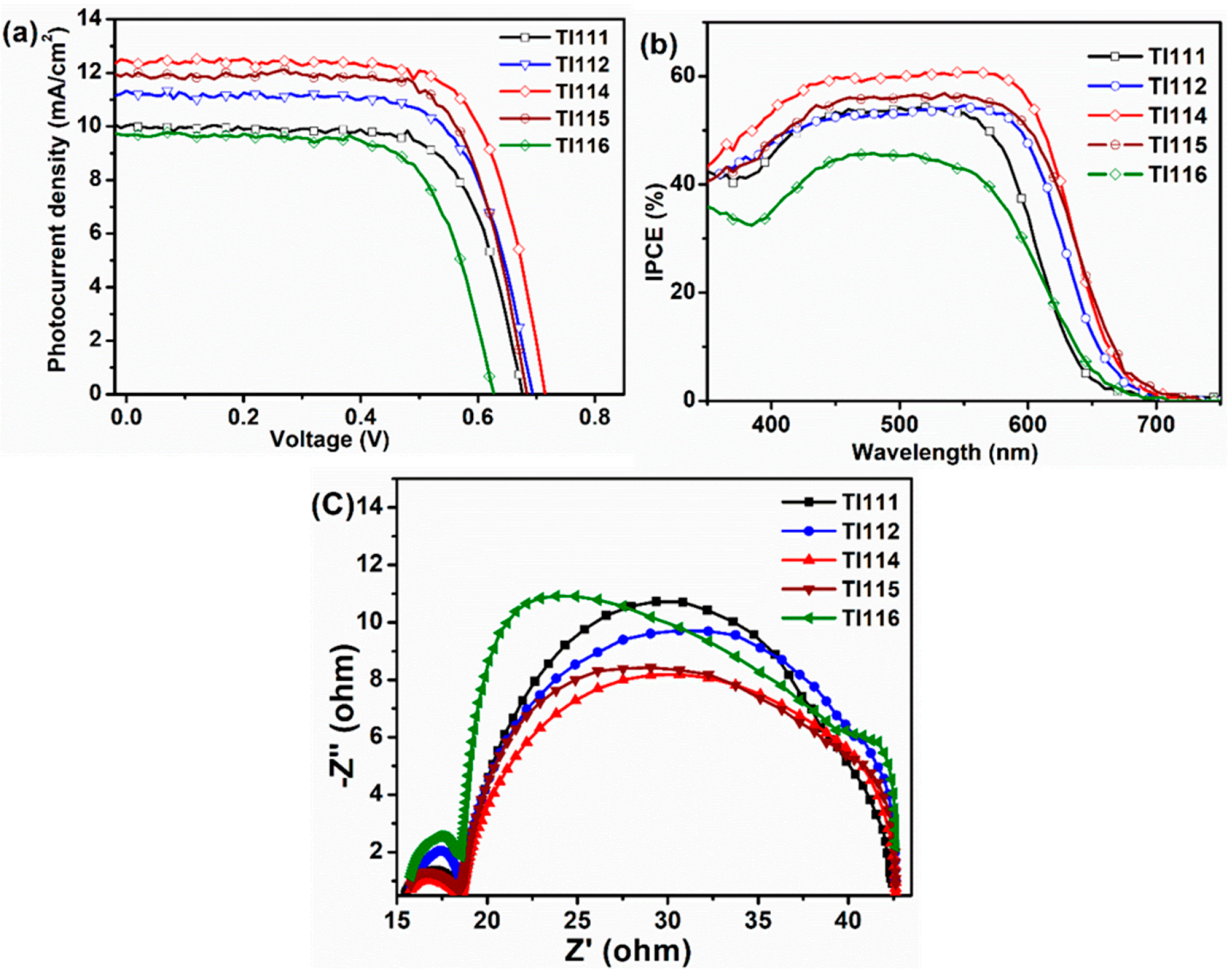
(**a**) Current versus potential plots and (**b**) Monochromatic IPCE spectra for sensitizers **TI111**–**TI116.** (**c**) EIS spectra measured under illumination of DSSCs based on **TI111**–**TI116** dyes.

Table 2 summarizes the cell performance of the DSSCs anchored with oxindole dyes: short-circuit photocurrent densities (*J*_sc_), open-circuit voltages (*V*_oc_), fill factors (FFs), and solar-to-electricity conversion efficiencies (η). Figure 4 presents their corresponding photocurrent density–voltage (*I*–*V*) curves, incident photo-to-current efficiency (IPCE) spectra and electrochemical impedance spectroscopy (EIS). The parent dye **TI111** exhibited *J*_sc_ = 10.03 mA cm^−2^, *V*_oc_ = 680 mV, and FF = 0.699, corresponding to an overall *η* = 4.76% (Table 2). Furthermore, incorporating the halogen substitutions in the oxindole dyes enhanced the device conversion efficiencies. The DSSC based on the F-substituted **TI112** sensitizer achieved *J*_sc_ = 11.32 mA cm^−2^, *V*_oc_ = 690 mV, and FF = 0.695, corresponding to an overall *η* = 5.43%, which was approximately 14% higher than that of the non-substituted oxindole sensitizer **TI111**. The enhanced *η* was due to its increase in the *J*_sc_ which could be attributed to the electronic coupling of F with the anchoring group [58]. Replacing the F with other halogen substituents in the oxindoles showed further enhancement in the DSSC performance. Among all the halogen substituted sensitizers reported in this study, the Br incorporated sensitizer **TI114** exhibited the highest photovoltaic parameters (*J*_sc_ = 12.46 mA cm^−2^, *V*_oc_ = 720 mV, and FF = 0.708) with an overall conversion efficiency (*η*) of 6.35%. The improved photocurrent of the **TI114** sensitizer suggests that compared to F substitution, the Br-substituted dye can exhibit better cell performance. Also, compared to the dye with the traditional cyanoacrylic acid acceptor bearing the same TPA donor-*π*-bridge moiety [67], the sensitizer with the Br-substituted oxindole acceptor seems to be more impressive. Surprisingly, changing the position of the substituent with respect to the anchoring group exhibited negative effect on the solar cell performance. The **TI116** anchored DSSC showed lower current density (*J*_sc_ = 9.66 mA cm^−2^, *V*_oc_ = 630 mV, and FF = 0.690), that is corresponding to an overall *η* = 4.21%, which may be due to the absence of electronic coupling of substitution with the anchoring group. In order to gain more insight into the electron transport of oxindole dyes, the electrochemical impedance of the cells was measured under illumination and is shown in Figure 4c. The fitted EIS data, the charge transfer resistance related to the recombination of electrons at the interface (R_k_), the electron transport resistance in the photoanode (R_w_), the effective rate constant for recombination (k_eff_), the electron lifetime in the photoanode (τ), the effective electron diffusion coefficient (D_eff_), and the electron diffusion length (L_n_) are compiled in Table S4 in SI. The radii of the semicircles in the EIS spectra of the DSSCs based on oxindole dyes, according to the Nyquist plot, is in the order of **TI116** > **TI111** > **TI112** > **TI115** > **TI114**. These results indicate that the charge transport is in the order of **TI114** > **TI115** > **TI112** > **TI111** > **TI116**, which agrees with the overall device performances. Also, the life time (**τ**) values shown in Table S4 indicates that the capacity to suppress the back reaction of the electron with the I_3_^−^ in the electrolyte are affected by the different halogen substituent on the oxindole moiety. The measured τ is in the order of **TI114** = **TI112** > **TI115** = **TI111** > **TI116** and is consistent with those of *V*_oc_ determined experimentally (see Table 2). Among all the sensitizers studied here, the dye **TI114** showed the highest charge transport, lowest electron transport resistance and better electron lifetime which have led enhancement in its *V*_oc_ and efficiency. The IPCE of the device using the **TI111** sensitizer exceeded 50% in the spectral region ranging from 425 to 565 nm and reached a maximum of 52% (Figure 4b). Incorporating halogen substituents in the sensitizers displayed red-shifted ranges and increasing IPCE maxima. The dye **TI114** with the Br substituent exhibited the maximum IPCE value of 60% at 585 nm, which was the highest among the listed oxindole dyes. Similar to the I–*V* curve (Figure 4a), altering the position of the substituent also resulted in a lower IPCE value as well as a blue shift for dye **TI116** (a maximum of 43% at 445 nm). Based on these analyses and the previous report [58], we can estimate that the halogen substituent, particularly positioned para to the anchoring group, can have strong impact on the device performance of the sensitizer. We believe that the effectiveness of electron transport, the proper balance of Fermi level, and controlling affinity of the acceptors towards the semiconductor are the crucial factors affecting device performances of oxindole-based DSSCs. Such factors cannot be predicted intuitively and their physical origins need to be further addressed in future work.

## 3. Materials and Methods

**General procedure for syntheses of compounds 3(a**–**d):** In a 100 mL round bottom flask 5-[4-(diphenylamino) phenyl] thiophene-2-carbaldehyde (**2**) (12 mmol) was treated with the appropriate oxindole (**1a**–**d**) (10 mmol) and piperidine (1 mmol) in 20 mL of ethanol. After the reaction mixture was refluxed for 6 h, it was cooled and the resulting precipitate was filtered, washed with cold ethanol, and dried to give the target compounds (70–90% yield). 

*(Z)-3-((5-(4-(Diphenylamino)phenyl)thiophen-2-yl)methylene)indolin-2-one* (3a; 3.8 g, 82.79%): ^1^H NMR (300 MHz, DMSO-*d*_6_) δ 10.64 (s, 1H); 8.05 (s, 1H); 7.89 (d, *J* = 4.2 Hz, 1H); 7.68 (d, *J* = 7.2 Hz, 1H); 7.66 (d, *J* = 8.7 Hz, 2H); 7.52 (d, *J* = 3.9 Hz, 1H); 7.35 (t, *J* = 7.8 Hz, 4H); 7.20 (t, *J* = 7.2 Hz, 1H); 7.14–7.08 (m, 6H); 7.02–6.97 (m, 3H); 6.88 (d, *J* = 7.8 Hz, 1H). ^13^C NMR (75 MHz, DMSO-*d*_6_) δ 167.8; 150.9; 148.1; 147.1; 140.8; 139.7; 136.4; 130.1; 128.7; 128.5; 127.5; 127.3; 125.1; 124.9; 124.2; 123.6; 122.9; 121.3; 121.3; 119.7; 109.9. HRMS (FAB) (*m*/*z* [M + H]^+^) calcd for C_31_H_23_N_2_OS, 471.1526; found 471.1513.

*(Z)-3-((5-(4-(Diphenylamino)phenyl)thiophen-2-yl)methylene)-5-fluoroindolin-2-one* (3b; 3.4 g, 70.69%): ^1^H NMR (300 MHz, DMSO-*d*_6_) δ 10.63 (s, 1H); 8.14 (s, 1H); 7.89 (d, *J* = 3.9 Hz, 1H);7.67 (d, *J* = 8.7 Hz, 2H); 7.60 (dd, *J* = 9, 2.4 Hz, 1H); 7.55 (d, *J* = 3.9 Hz, 1H); 7.37 (t, *J* = 7.6 Hz, 4H); 7.14 (d, *J* = 7.2 Hz, 2H); 7.10 (d, *J* = 8.1 Hz, 5H); 7.01 (d, *J* = 8.4 Hz, 2H); 6.84 (dd, *J* = 8.4, 4.5 Hz, 1H). ^13^C NMR (75 MHz, DMSO-*d*_6_) δ 167.9; 159.8; 156.7; 151.7; 148.2; 147.0; 140.3; 137.0; 136.1; 130.1; 127.4; 127.3; 126.5; 126.4; 125.1; 124.2; 123.7; 122.8; 120.8 (d, *J* = 7.5 Hz); 114.7 (d, *J* = 22.5 Hz); 110.4 (d, *J* = 7.5 Hz); 107 (d, *J* = 24.7 Hz). HRMS (FAB) (*m*/*z* [M]^+^) calcd for C_31_H_21_FN_2_OS, 488.1353; found 488.1350.

*(Z)-5-bromo-3-((5-(4-(Diphenylamino)phenyl)thiophen-2-yl)methylene)indolin-2-one* (3c; 4.2 g, 76.45%): [54]**:** ^1^H NMR (300 MHz, DMSO-*d*_6_) δ 10.75 (s, 1H); 8.22 (s, 1H); 7.93 (s, 1H); 7.89 (d, *J* = 3.6 Hz, 1H); 7.69 (d, *J* = 8.7 Hz, 2H); 7.57 (d, *J* = 3.9 Hz, 1H); 7.40–7.35 (m, 5H); 7.16–7.10 (m, 6H); 7.02 (d, *J* = 8.4 Hz, 2H); 6.83 (d, *J* = 8.4 Hz, 1H). ^13^C NMR (75 MHz, DMSO-*d*_6_) δ 167.4; 151.9; 148.3; 147.0; 140.6; 139.7; 136.2; 130.7; 130.4; 130.2; 127.5; 127.3; 127.2; 125.1; 124.3; 123.8; 122.7; 122.4; 119.8; 113.3; 111.7. HRMS (FAB) (*m*/*z* [M + H]^+^) calcd for C_31_H_22_BrN_2_OS, 549.0631; found 549.0620.

*(Z)-3-((5-(4-(Diphenylamino)phenyl)thiophen-2-yl)methylene)-5-iodoindolin-2-one* (3d; 5.2 g, 86.95%): ^1^H NMR (300 MHz, DMSO-*d*_6_) δ 10.71 (s, 1H); 8.19 (s, 1H); 8.07 (d, *J* = 1.5 Hz, 1H); 7.90 (d, *J* = 3.9 Hz, 1H); 7.68 (d, *J* = 8.4 Hz, 2H); 7.56 (d, *J* = 3.9 Hz, 1H); 7.51 (dd, *J* = 8.1, 1.5 Hz, 1H); 7.37 (t, *J* = 7.8 Hz, 4H); 7.15 (d, *J* = 7.5 Hz, 2H); 7.11 (d, *J* = 7.5 Hz, 4H); 7.02 (d, *J* = 8.4 Hz, 2H); 6.72 (d, *J* = 8.1 Hz, 1H). ^13^C NMR (75 MHz, DMSO-*d*_6_) δ 167.2; 151.8; 148.2; 147.0; 140.5; 140.1; 136.5; 136.2; 130.1; 127.9; 127.7; 127.4; 127.2; 125.1; 124.2; 123.7; 122.8; 119.6; 112.2; 84.3. HRMS (FAB) (*m*/*z* [M]^+^) calcd for C_31_H_21_IN_2_OS, 596.0414; found 596.0413.

**General procedure for syntheses of compounds 4(a**–**d):** To a solution of compound **3(a**–**d)** (10 mmol) in dimethylformamide (50 mL), potassium carbonate (12 mmol) was added and stirred for 30 min. Methyl bromoacetate (12 mmol) and tetrabutylammonium bromide (1 mmol) were then added and reaction mixture was continued stirring at room temperature for 48 h. The reaction mixture was diluted by ethyl acetate (30 mL) and the salt formed was removed by filtration and washed with ethyl acetate (6 mL). The solution was then concentrated under reduced pressure. The resulting residue was purified by column chromatography (SiO_2_; CH_2_Cl_2_) to yield ester compounds (60–65% yield).

*Methyl (Z)-2-(3-((5-(4-(diphenylamino)phenyl)thiophen-2-yl)methylene)-2-oxoindolin-1-yl)acetate* (4a; 2.81 g, 64.15%): ^1^H NMR (300 MHz, DMSO-*d*_6_) δ 8.17 (s, 1H); 7.89 (d, *J* = 4.2 Hz, 1H); 7.75 (d, *J* = 6.9 Hz, 1H); 7.67 (d, *J* = 8.7 Hz, 2H); 7.56 (d, *J* = 3.9 Hz, 1H); 7.35 (t, *J* = 7.8 Hz, 4H); 7.25 (t, *J* = 7.9 Hz, 1H) 7.14–7.09 (m, 7H); 7.06 (d, *J* = 8.1 Hz, 1H); 7.01 (d, *J* = 5.4 Hz, 2H); 4.69 (s, 2H); 3.69 (s, 3H). ^13^C NMR (75 MHz, CDCl_3_) δ 168.5; 166.3; 152.5; 148.3; 147.2; 140.4; 139.1; 136.2; 129.4; 129.0; 128.0; 127.4; 127.1; 124.9; 124.1; 123.5; 122.9; 122.5; 122.1; 119.2; 118.7; 107.9; 52.5; 41.0. HRMS (FAB) (*m*/*z* [M + H]^+^) calcd for C_34_H_27_N_2_O_3_S, 543.1737; found 543.1732.

*Methyl (Z)-2-(3-((5-(4-(diphenylamino)phenyl)thiophen-2-yl)methylene)-5-fluoro-2-oxoindolin-1-yl)acetate* (4b; 2.39 g, 61.43%): ^1^H NMR (300 MHz, DMSO-*d*_6_) δ 8.29 (s, 1H); 7.91 (d, *J* = 4.2 Hz, 1H); 7.71 (d, *J* = 8.7 Hz, 2H); 7.61 (d, *J* = 3.9 Hz, 1H); 7.38 (t, *J* = 7.6 Hz, 4H); 7.15-7.10 (m, 9H); 7.10 (d, *J* = 3.9 Hz, 2H); 4.72 (s, 2H); 3.72 (s, 3H). ^13^C NMR (75 MHz, CDCl_3_) δ 168.4; 166.2; 161.2; 159.8; 153.4; 148.4; 147.1; 139.8; 136.3; 135.8; 130.1; 129.4; 127.1; 124.9; 123.6; 122.7; 122.6; 118.6; 114.1 (d, *J* = 22.5 Hz); 108.3 (d, *J* = 7.5 Hz); 106.1 (d, *J* = 22.5 Hz); 52.5; 41.1. HRMS (FAB) (*m*/*z* [M]^+^) calcd for C_34_H_25_FN_2_O_3_S, 560.1564; found 560.1568.

*Methyl (Z)-2-(5-bromo-3-((5-(4-(diphenylamino)phenyl)thiophen-2-yl)methylene)-2-oxoindolin-1-yl)acetate* (4c; 3.02 g, 63.84%) ^1^H NMR (300 MHz, DMSO-*d*_6_) δ 8.35 (s, 1H); 8.03 (s, 1H); 7.91 (d, *J* = 3.6 Hz, 1H); 7.70 (d, *J* = 8.4 Hz, 2H); 7.60 (d, *J* = 3.9 Hz, 1H) 7.44 (d, *J* = 8.4 Hz, 1H); 7.38 (t, *J* = 7.6 Hz, 4H); 7.17–7.10 (m, 6H); 7.06 (d, *J* = 8.4 Hz, 1H); 7.01 (d, *J* = 8.4 Hz, 2H); 4.72 (s, 2H); 3.71 (s, 3H). ^13^C NMR (75 MHz, CDCl_3_) δ 168.3; 165.8; 153.5; 148.4; 140.1; 139.0; 135.8; 130.2; 130.2; 129.4; 127.1; 126.1; 125.0; 123.6; 122.7; 121.6; 117.6; 114.8; 109.3. HRMS (FAB) (*m*/*z* [M + H]^+^) calcd for C_34_H_26_BrN_2_O_3_S, 621.0842; found 621.0841.

*Methyl (Z)-2-(3-((5-(4-(diphenylamino)phenyl)thiophen-2-yl)methylene)-5-iodo-2-oxoindolin-1-yl)acetate* (4d; 4.63 g, 64.84%) ^1^H NMR (300 MHz, DMSO-*d*_6_) δ 8.28 (s, 1H); 8.13 (s, 1H); 7.89 (d, *J* = 3.9 Hz, 1H); 7.66 (d, *J* = 8.7 Hz, 2H); 7.57 (d, *J* = 8.4 Hz, 1H); 7.55 (d, *J* = 3.9 Hz, 1H); 7.36 (t, *J* = 7.8 Hz, 4H); 7.15–7.08 (m, 6H); 6.99 (d, *J* = 8.7 Hz, 2H); 6.92 (d, *J* = 8.4 Hz, 1H); 4.69 (s, 2H); 3.71 (s, 3H). ^13^C NMR (75 MHz, DMSO-*d*_6_) δ 169.0; 165.5; 152.5; 148.4; 147.0; 141.2; 140.3; 136.4; 136.1; 131.3; 130.1; 127.8; 127.4; 127.0; 126.6; 125.2; 124.3; 123.8; 122.6; 117.6; 111.5; 85.4; 52.7; 41.2. HRMS (FAB) (*m/z* [M]^+^) calcd for C_34_H_25_IN_2_O_3_S, 668.0625; found 668.0622.

**General procedure for syntheses of sensitizers TI111**–**TI115:** Compound **4(a**–**d)** (9 mmol) was dissolved in THF (6 mL) and 0.5 M NaOH_(aq.)_ (18 mmol) was added to the solution, which was then stirred at room temperature for 6 h. After the hydrolysis was completed, the solvent was evaporated. Ethanol (5 mL) was added and the resulting precipitate was filtered, washed with cold ethanol (0.5 mL), and dried. The precipitate were then dissolved in water (10 mL) and 0.5 M HCl_(aq.)_ was added to precipitate the product. The solid was filtered, washed with water (3 × 6 mL) and dried under vacuum to afford target sensitizers (80–90% yield).

Dye **TI111** (2.35 g, 86.08%): ^1^H NMR (500 MHz, DMSO-*d*_6_) δ 8.32 (d, *J* = 7.6 Hz, 1H); 7.85 (s, 2H); 7.73 (d, *J* = 8.6 Hz, 2H); 7.60 (d, *J* = 4 Hz, 1H); 7.37–7.32 (m, 5H); 7.14–7.05 (m, 8H); 6.99 (d, *J* = 8.5 Hz, 2H); 4.54 (s, 2H). ^13^C NMR (125 MHz, DMSO-*d*_6_) δ 169.9; 168.6; 150.0; 148.4; 146.9; 143.1; 139.4; 135.5; 130.1; 129.8; 128.5; 127.6; 126.5; 125.2; 124.4; 124.3; 123.6; 122.6; 122.4; 121.2; 120.6; 109.4; 41.6. HRMS (FAB) (*m/z* [M + H]^+^) calcd for C_33_H_25_N_2_O_3_S, 529.1580; found 529.1570.

Dye **TI112** (2.38 g, 80.13%): ^1^H NMR (300 MHz, DMSO-*d*_6_) δ 8.24 (s, 1H); 7.91(d, *J* = 3.9 Hz, 1H); 7.71 (d, *J* = 8.7 Hz, 2H); 7.66 (d, *J* = 2.1 Hz, 1H); 7.59 (d, *J* = 3.9 Hz, 1H); 7.38 (t, *J* = 7.6 Hz, 4H); 7.17–7.11 (m, 8H); 7.02 (d, *J* = 8.7 Hz, 2H); 4.52 (s, 2H). ^13^C NMR (125 MHz, DMSO-*d*_6_) δ 169.8; 168.4; 159.1; 157.3; 150.8; 148.5; 146.9; 140.2; 139.4; 135.0; 130.1; 127.6; 126.2; 125.2; 124.6; 124.4; 122.4; 121.5; (d, *J* = 8.8 Hz); 120.5; 115.9 (d, *J* = 40 Hz); 110.5 (d, *J* = 41.2 Hz); 41.7. HRMS (FAB) (*m/z* [M]^+^) calcd for C_33_H_23_FN_2_O_3_S, 546.1408; found 546.1405.

Dye **TI114** (2.63 g, 89.15%): ^1^H NMR (500 MHz, DMSO-*d*_6_) δ 8.29 (s, 1H); 7.87 (s, 2H); 7.64 (d, *J* = 8.2 Hz, 2H); 7.59 (s, 1H); 7.49 (d, *J* = 8 Hz, 1H); 7.34 (t, *J* = 7.3 Hz, 4H); 7.13–7.04 (m, 7H); 6.97 (d, *J* = 8.2 Hz, 2H); 4.53 (s, 2H). ^13^C NMR (125 MHz, DMSO-*d*_6_) δ 169.7; 168.1; 150.7; 148.7; 146.9; 142.2; 140.3; 135.0; 132.0; 130.2; 127.6; 126.1; 125.3; 124.8; 124.4; 122.7; 122.4; 120.0; 113.9; 111.4; 41.7. HRMS (FAB) (*m*/*z* [M]^+^) calcd for C_33_H_23_BrN_2_O_3_S, 606.0613; found 606.0603.

Dye **TI115** (3.23 g, 87.53%): ^1^H NMR (500 MHz, DMSO-*d*_6_) δ 8.28 (s, 1H); 8.11 (s, 1H); 7.88 (d, *J* = 3.7 Hz, 1H); 7.68 (d, *J* = 8.5 Hz, 2H); 7.56 (d, *J* = 3.7 Hz, 1H); 7.34 (d, *J* = 7.3 Hz, 4H); 7.11-7.08 (m, 7H); 6.98 (d, *J* = 3.7 Hz, 2H); 6.89 (d, *J* = 8.2 Hz, 1H); 4.53 (s, 2H). ^13^C NMR (125 MHz, DMSO-*d*_6_) δ 169.9; 165.5; 152.3; 148.3; 147.0; 141.0; 140.7; 136.3; 136.1; 131.0; 130.1; 127.7; 127.4; 127.1; 126.5; 125.3; 125.2; 124.3; 123.8; 122.6; 117.9; 111.5; 85.2; 41.5. HRMS (FAB) (*m/z* [M]^+^) calcd for C_33_H_23_IN_2_O_3_S, 654.0469; found 654.0467.

*(Z)-6-bromo-3-((5-(4-(Diphenylamino)phenyl)thiophen-2-yl)methylene)indolin-2-one* (**6**): 6-Bromo-1H-indole-2,3-dione (**5**) (2 g, 8.84 mmol), 5-[4-(diphenylamino)phenyl]thiophene-2-carbaldehyde (**2**) (6.29 g, 17.69 mmol), and PPh_3_ (4.64 g, 17.69 mmol) were combined and dissolved in 20 mL of toluene in a microwave reaction vessel containing a magnetic stir bar. The reaction vessel was irradiated in the Synthos 3000 microwave oven at 300 W for 4 h (up to 110 °C). After the removal of solvent, the crude product was purified by column chromatography (SiO_2_; CH_2_Cl_2_) to afford compound **6** (2.88, 60% yield). ^1^H NMR (300 MHz, DMSO-*d*_6_) δ 10.76 (s, 1H); 8.13 (s, 1H); 7.91 (d, *J* = 3.6 Hz, 1H); 7.68 (d, *J* = 7.8 Hz, 1H); 7.64 (d, *J* = 8.4 Hz, 2H); 7.55 (d, *J* = 3.9 Hz, 1H); 7.37 (t, *J* = 7.5 Hz, 4H); 7.19 (d, *J* = 8.1, 1H); 7.16-7.09 (m, 6H); 7.03–7.00 (m, 3H). ^13^C NMR (75 MHz, DMSO-*d*_6_) δ 167.5; 151.6; 148.2; 147.0; 141.9; 140.4; 136.2; 130.2; 129.7; 127.4; 127.3; 125.1; 124.3; 24.0; 123.8; 122.8; 121.4; 120.9; 120.0; 112.5. HRMS (FAB) (*m/z* [M + H]^+^) calcd for C_31_H_22_ON_2_BrS, 549.0631; found 549.0632.

*Methyl (Z)-2-(6-bromo-3-((5-(4-(diphenylamino)phenyl)thiophen-2-yl)methylene)-2-oxoindolin-1-yl)acetate* (**7**): Compound **7** was prepared from compound **6** using a method similar to that described for the synthesis of compound **4** to get 1.86 g (57%) final ester. ^1^H NMR (300 MHz, DMSO-*d*_6_) δ 8.23 (s, 1H); 7.90 (d, *J* = 3.6 Hz, 1H); 7.69 (d, *J* = 7.2 Hz, 1H); 7.67 (d, *J* = 7.8 Hz, 2H); 7.56 (d, *J* = 3.9 Hz, 1H); 7.39–7.32 (m, 5H); 7.27 (d, *J* = 8.1 Hz, 1H); 7.14–7.07 (m, 6H); 6.98 (d, *J* = 7.8 Hz, 2H); 4.71 (s, 2H); 3.69 (s, 3H). ^13^C NMR (75 MHz, CDCl_3_) δ 168.1; 166.0; 153.2; 148.4; 147.1; 141.2; 139.5; 136.0; 129.5; 129.3; 127.2; 127.0; 124.9; 123.5; 123.1; 122.7; 122.6; 121.3; 119.6; 118.0; 111.2; 52.6; 41.0. HRMS (FAB) (*m/z* [M]^+^) calcd for C_34_H_25_BrN_2_O_3_S, 620.0769; found 620.0770.

Synthesis of sensitizer **TI116**: Dye **TI116** was prepared from compound **7** using a method similar to that described for the synthesis of sensitizer **TI111** to get 1.03 g (56%) ^1^H NMR (500 MHz, DMSO-*d*_6_) δ 8.22 (d, *J* = 8.1 Hz, 1H); 7.89 (d, *J* = 4.2 Hz, 2H); 7.73 (d, *J* = 8.7 Hz, 2H); 7.61 (d, *J* = 3.9 Hz, 1H); 7.40–7.28 (m, 6H); 7.14–7.08 (m, 6H); 6.98 (d, *J* = 8.4 Hz, 2H); 4.56 (s, 2H). ^13^C NMR (125 MHz, DMSO-*d*_6_) δ 169.8; 168.5; 150.6; 148.5; 146.9; 144.5; 140.0; 135.3; 130.1; 129.3; 127.6; 126.4; 125.2; 124.9; 124.6; 124.4; 122.6; 122.4; 119.9; 119.8; 112.6; 41.8. HRMS (FAB) (*m*/*z* [M]^+^) calcd for C_33_H_23_BrN_2_O_3_S, 606.0607; found 606.0606.

## 4. Conclusions

In conclusion, we have designed and synthesized new acceptors bearing halogen-substituted oxindoles for the application in DSSCs. Among all the halogen-substituted acceptors studied in this artical, the sensitizer with Br exhibited superior photo-physical properties. Also, the amide carbonyl groups of these acceptors can have partial chelation to the TiO_2_ surface providing an added benefit towards effective electron injection. The new oxindole building block can provided a new gateway for future rational dye design for applications in DSSC. Evidently, further enhancement in the absorption profiles of oxindole sensitizers can be obtained by using different electron withdrawing substituents and adding efficient donating moieties. Such structural modification of oxindole-based sensitizers to gain superior performances in DSSC is currently underway in our laboratory.

## Figures and Tables

**Table 1:** Electrochemical properties for **TI111**–**TI116** dyes.

Dye	λ_abs_/nm (ε/L mol^−1^ cm^−1^)	*E*_HOMO_[a] (eV)	*E*_LUMO_[b] (eV)	*E*_0-0_[b] (eV)
**TI111**	469 (3.7737)	5.11	3.02	2.09
**TI112**	480 (3.0562)	5.11	3.05	2.06
**TI114**	483 (3.8825)	5.11	3.08	2.03
**TI115**	483 (3.4712)	5.12	3.09	2.03
**TI116**	410 (1.7437)	5.11	3.06	2.05

[a] *E*_HOMO_ = *E*_ox_ − *E*_Fc/Fc_^+^ + 4.8 eV. [b] *E*_LUMO_ = *E*_HOMO_ – *E*_0-0_. The band gap, *E*_0-0_, was estimated from the onset of absorption spectrum measured in CH_2_Cl_2_.

**Table 2:** Photovoltaic parameters of devices incorporating **TI111**-**TI116** sensitizers.

Sensitizer	*J_SC_* (mA cm^−2^)	*V_OC_* (mV)	*FF*	*η* (%)
**TI111**	10.03	680	0.669	4.76
**TI112**	11.32	690	0.695	5.43
**TI114**	12.46	720	0.708	6.35
**TI115**	11.97	680	0.714	5.81
**TI116**	9.66	630	0.692	4.21

## Supplementary Materials

The following are available online. Figure S1: Theoretical calculation for the location of HOMOs and LUMOs, Table S1: Bond lengths [Å] and angles [°] for ester **4b**, Table S2: Bond lengths [Å] and angles [°] for ester **7**.

## References

[1] Grätzel M. (2005). Mesoscopic solar cells for electricity and hydrogen production from sunlight. Chem. Lett..

[2] Yun S., Lund P.D., Hinsch A. (2015). Stability assessment of alternative platinum free counter electrodes for dye-sensitized solar cells. Energy Environ. Sci..

[3] Wu J., Lan Z., Lin J., Huang M., Huang Y., Fan L., Luo G. (2015). Electrolytes in Dye-Sensitized Solar Cells. Chem. Rev..

[4] Sugathan V., John E., Sudhakar K.J.R., Reviews S.E. (2015). Recent improvements in dye sensitized solar cells: A review. Renew. Sustain. Energy Rev..

[5] Docampo P., Guldin S., Leijtens T., Noel N.K., Steiner U., Snaith H.J. (2014). Lessons Learned: From Dye-Sensitized Solar Cells to All-Solid-State Hybrid Devices. Adv. Mater..

[6] Zhang S., Yang X., Numata Y., Han L. (2013). Highly efficient dye-sensitized solar cells: Progress and future challenges. Energy Environ. Sci..

[7] Grätzel C., Zakeeruddin S.M. (2013). Recent trends in mesoscopic solar cells based on molecular and nanopigment light harvesters. Mater. Today.

[8] Hardin B.E., Snaith H.J., McGehee M.D. (2012). The renaissance of dye-sensitized solar cells. Nat. Photon..

[9] Hagfeldt A., Boschloo G., Sun L., Kloo L., Pettersson H. (2010). Dye-Sensitized Solar Cells. Chem. Rev..

[10] Mishra A., Fischer M.K.R., Bäuerle P. (2009). Metal-Free Organic Dyes for Dye-Sensitized Solar Cells: From Structure: Property Relationships to Design Rules. Angew. Chem. Int. Ed..

[11] Robertson N. (2006). Optimizing Dyes for Dye-Sensitized Solar Cells. Angew. Chem. Int. Ed..

[12] Nazeeruddin M.K., Grätzel M., McCleverty J.A., Meyer T.J. (2003). Comprehensive Coordination Chemistry II.

[13] Grätzel M. (2003). Dye-sensitized solar cells. J. Photochem. Photobiol. C Photochem. Rev..

[14] Grätzel M. (2001). Photoelectrochemical cells. Nature.

[15] O’Regan B., Grätzel M. (1991). A low-cost, high-efficiency solar cell based on dye-sensitized colloidal TiO2 films. Nature.

[16] Maddah H.A., Berry V., Behura S.K. (2020). Biomolecular photosensitizers for dye-sensitized solar cells: Recent developments and critical insights. Renew. Sustain. Energy Rev..

[17] Boschloo G. (2019). Improving the performance of dye-sensitized solar cells. Front. Chem..

[18] Sharma K., Sharma V., Sharma S.S. (2018). Dye-Sensitized Solar Cells: Fundamentals and Current Status. Nanoscale Res. Lett..

[19] Yin J.-F., Velayudham M., Bhattacharya D., Lin H.-C., Lu K.-L. (2012). Structure optimization of ruthenium photosensitizers for efficient dye-sensitized solar cells—A goal toward a “bright” future. Coord. Chem. Rev..

[20] Robson K.C.D., Bomben P.G., Berlinguette C.P. (2012). Cycloruthenated sensitizers: Improving the dye-sensitized solar cell with classical inorganic chemistry principles. Dalton Trans..

[21] Nazeeruddin M.K., Kay A., Rodicio I., Humphry-Baker R., Müller E., Liska P., Vlachopoulos N., Grätzel M. (1993). Conversion of light to electricity by cis-X2bis(2,2’-bipyridyl-4,4’-dicarboxylate)ruthenium(II) charge-transfer sensitizers (X = Cl-, Br-, I-, CN-, and SCN-) on nanocrystalline titanium dioxide electrodes. J. Am. Chem. Soc..

[22] Nazeeruddin M.K., Péchy P., Renouard T., Zakeeruddin S.M., Humphry-Baker R., Comte P., Liska P., Cevey L., Costa E., Shklover V. (2001). Engineering of Efficient Panchromatic Sensitizers for Nanocrystalline TiO2-Based Solar Cells. J. Am. Chem. Soc..

[23] Nazeeruddin M.K., Splivallo R., Liska P., Comte P., Grätzel M. (2003). A swift dye uptake procedure for dye sensitized solar cells. Chem. Commun..

[24] Grätzel M.J.J.o.P., Chemistry P.A. (2004). Conversion of sunlight to electric power by nanocrystalline dye-sensitized solar cells. J. Photochem. Photobiol. A.

[25] Nazeeruddin M.K., De Angelis F., Fantacci S., Selloni A., Viscardi G., Liska P., Ito S., Takeru B., Grätzel M. (2005). Combined Experimental and DFT-TDDFT Computational Study of Photoelectrochemical Cell Ruthenium Sensitizers. J. Am. Chem. Soc..

[26] Yasuo C., Ashraful I., Yuki W., Ryoichi K., Naoki K., Liyuan H. (2006). Dye-Sensitized Solar Cells with Conversion Efficiency of 11.1%. Jpn. J. Appl. Phys. Part 1.

[27] Wang S.-W., Chou C.-C., Hu F.-C., Wu K.-L., Chi Y., Clifford J.N., Palomares E., Liu S.-H., Chou P.-T., Wei T.-C. (2014). Panchromatic Ru(ii) sensitizers bearing single thiocyanate for high efficiency dye sensitized solar cells. J. Mater. Chem. A.

[28] Jia H.-L., Li S.-S., Gong B.-Q., Gu L., Bao Z.-L., Guan M.-Y. (2020). Efficient cosensitization of new organic dyes containing bipyridine anchors with porphyrins for dye-sensitized solar cells. Sustain. Energy Fuels.

[29] Higashino T., Imahori H. (2015). Porphyrins as excellent dyes for dye-sensitized solar cells: Recent developments and insights. Dalton Trans..

[30] Yella A., Mai C.-L., Zakeeruddin S.M., Chang S.-N., Hsieh C.-H., Yeh C.-Y., Grätzel M. (2014). Molecular Engineering of Push–Pull Porphyrin Dyes for Highly Efficient Dye-Sensitized Solar Cells: The Role of Benzene Spacers. Angew. Chem. Int. Ed..

[31] Urbani M., Grätzel M., Nazeeruddin M.K., Torres T. (2014). Meso-Substituted Porphyrins for Dye-Sensitized Solar Cells. Chem. Rev..

[32] Li L.-L., Diau E.W.-G. (2013). Porphyrin-sensitized solar cells. Chem. Soc. Rev..

[33] Bessho T., Zakeeruddin S.M., Yeh C.-Y., Diau E.W.-G., Grätzel M. (2010). Highly Efficient Mesoscopic Dye-Sensitized Solar Cells Based on Donor–Acceptor-Substituted Porphyrins. Angew. Chem. Int. Ed..

[34] Collavini S., Völker S.F., Delgado J.L. (2015). Understanding the Outstanding Power Conversion Efficiency of Perovskite-Based Solar Cells. Angew. Chem. Int. Ed..

[35] Zhao Y., Zhu K. (2016). Organic-inorganic hybrid lead halide perovskites for optoelectronic and electronic applications. Chem. Soc. Rev..

[36] Green M.A., Ho-Baillie A., Snaith H.J. (2014). The emergence of perovskite solar cells. Nat. Photonics.

[37] Rong Y., Liu L., Mei A., Li X., Han H. (2015). Beyond Efficiency: The Challenge of Stability in Mesoscopic Perovskite Solar Cells. Adv. Energy Mater..

[38] Yen Y.-S., Chou H.-H., Chen Y.-C., Hsu C.-Y., Lin J.T. (2012). Recent developments in molecule-based organic materials for dye-sensitized solar cells. J. Mater. Chem..

[39] Yao Z., Zhang M., Wu H., Yang L., Li R., Wang P. (2015). Donor/Acceptor Indenoperylene Dye for Highly Efficient Organic Dye-Sensitized Solar Cells. J. Am. Chem. Soc..

[40] Wu Y., Zhu W. (2013). Organic sensitizers from D-[small pi]-A to D-A-[small pi]-A: Effect of the internal electron-withdrawing units on molecular absorption, energy levels and photovoltaic performances. Chem. Soc. Rev..

[41] Kanaparthi R.K., Kandhadi J., Giribabu L. (2012). Metal-free organic dyes for dye-sensitized solar cells: Recent advances. Tetrahedron.

[42] Ooyama Y., Harima Y. (2009). Molecular Designs and Syntheses of Organic Dyes for Dye-Sensitized Solar Cells. Eur. J. Org. Chem..

[43] Cong J., Yang X., Liu J., Zhao J., Hao Y., Wang Y., Sun L. (2012). Nitro group as a new anchoring group for organic dyes in dye-sensitized solar cells. Chem. Commun..

[44] Rice C.R., Ward M.D., Nazeeruddin M.K., Grätzel M. (2000). Catechol as an efficient anchoring group for attachment of ruthenium-polypyridine photosensitisers to solar cells based on nanocrystalline TiO2 films. New J. Chem..

[45] Nazeeruddin M.K., Zakeeruddin S.M., Humphry-Baker R., Jirousek M., Liska P., Vlachopoulos N., Shklover V., Fischer C.-H., Grätzel M. (1999). Acid−Base Equilibria of (2,2‘-Bipyridyl-4,4‘-dicarboxylic acid)ruthenium(II) Complexes and the Effect of Protonation on Charge-Transfer Sensitization of Nanocrystalline Titania. Inorg. Chem..

[46] Numata Y., Ashraful I., Shirai Y., Han L. (2011). Preparation of donor-acceptor type organic dyes bearing various electron-withdrawing groups for dye-sensitized solar cell application. Chem. Commun..

[47] Delcamp J.H., Yella A., Nazeeruddin M.K., Grätzel M. (2012). Modulating dye E(S+/S*) with efficient heterocyclic nitrogen containing acceptors for DSCs. Chem. Commun..

[48] Hong J., Lai H., Liu Y., Yuan C., Li Y., Liu P., Fang Q. (2013). New organic dyes containing E- or Z-trifluoromethyl acrylic acid as the electron acceptors for dye-sensitized solar cell applications: An investigation of the effect of molecular configuration on the power conversion efficiency of the cells. RSC Adv..

[49] Li T.-Y., Su C., Akula S.B., Sun W.-G., Chien H.-M., Li W.-R. (2016). New Pyridinium Ylide Dyes for Dye Sensitized Solar Cell Applications. Org. Lett..

[50] Silva J.F.M., Garden S.J., Pinto A.C. (2001). The chemistry of isatins: A review from 1975 to 1999. J. Braz. Chem. Soc..

[51] Sun L., Tran N., Tang F., App H., Hirth P., McMahon G., Tang C. (1998). Synthesis and Biological Evaluations of 3-Substituted Indolin-2-ones:  A Novel Class of Tyrosine Kinase Inhibitors That Exhibit Selectivity toward Particular Receptor Tyrosine Kinases. J. Med. Chem..

[52] Marfat A., Robinson R.P. (1998). Preparation of azaoxindole-1-carboxamides as antiinflammatories and analgesics. U.S. Patent.

[53] Robinson R.R., Donahue K.M., Son P.S., Wagy S.D. (1996). Synthesis of substituted azaoxindoles for the preparation of aza-tenidap analogs. J. Heterocyclic Chem..

[54] Tingare Y.S., Shen M.-T., Su C., Ho S.-Y., Tsai S.-H., Chen B.-R., Li W.-R. (2013). Novel Oxindole Based Sensitizers: Synthesis and Application in Dye-Sensitized Solar Cells. Org. Lett..

[55] Simon S.J.C., Parlane F.G.L., Swords W.B., Kellett C.W., Du C., Lam B., Dean R.K., Hu K., Meyer G.J., Berlinguette C.P. (2016). Halogen Bonding Promotes Higher Dye-Sensitized Solar Cell Photovoltages. J. Am. Chem. Soc..

[56] Yoder N.C., Kumar K. (2002). Fluorinated amino acids in protein design and engineering. Chem. Soc. Rev..

[57] Babudri F., Farinola G.M., Naso F., Ragni R. (2007). Fluorinated organic materials for electronic and optoelectronic applications: The role of the fluorine atom. Chem. Commun..

[58] Chen B.-S., Chen D.-Y., Chen C.-L., Hsu C.-W., Hsu H.-C., Wu K.-L., Liu S.-H., Chou P.-T., Chi Y. (2011). Donor-acceptor dyes with fluorine substituted phenylene spacer for dye-sensitized solar cells. J. Mater. Chem..

[59] Bhim Raju T., Vaghasiya J.V., Afroz M.A., Soni S.S., Iyer P.K. (2016). Design, synthesis and DSSC performance of o-fluorine substituted phenylene spacer sensitizers: Effect of TiO2 thickness variation. Phys. Chem. Chem. Phys..

[60] Jie J., Xu Q., Yang G., Feng Y., Zhang B. (2020). Porphyrin sensitizers involving a fluorine-substituted benzothiadiazole as auxiliary acceptor and thiophene as π bridge for use in dye-sensitized solar cells (DSSCs). Dyes Pigments.

[61] Mao J., He N., Ning Z., Zhang Q., Guo F., Chen L., Wu W., Hua J., Tian H. (2012). Stable Dyes Containing Double Acceptors without COOH as Anchors for Highly Efficient Dye-Sensitized Solar Cells. Angew. Chem..

[62] Mann J.R., Gannon M.K., Fitzgibbons T.C., Detty M.R., Watson D.F. (2008). Optimizing the Photocurrent Efficiency of Dye-Sensitized Solar Cells through the Controlled Aggregation of Chalcogenoxanthylium Dyes on Nanocrystalline Titania Films. J. Phys. Chem. C.

[63] Kuang D., Uchida S., Humphry-Baker R., Zakeeruddin S.M., Grätzel M. (2008). Organic Dye-Sensitized Ionic Liquid Based Solar Cells: Remarkable Enhancement in Performance through Molecular Design of Indoline Sensitizers. Angew. Chem. Int. Ed..

[64] Tian H., Yang X., Chen R., Pan Y., Li L., Hagfeldt A., Sun L. (2007). Phenothiazine derivatives for efficient organic dye-sensitized solar cells. Chem. Commun..

[65] Chen R., Yang X., Tian H., Sun L. (2007). Tetrahydroquinoline dyes with different spacers for organic dye-sensitized solar cells. J. Photochem. Photobiol. A.

[66] Hara K., Wang Z.-S., Sato T., Furube A., Katoh R., Sugihara H., Dan-oh Y., Kasada C., Shinpo A., Suga S. (2005). Oligothiophene-Containing Coumarin Dyes for Efficient Dye-Sensitized Solar Cells. J. Phys. Chem. B.

[67] Liu W.-H., Wu I.C., Lai C.-H., Lai C.-H., Chou P.-T., Li Y.-T., Chen C.-L., Hsu Y.-Y., Chi Y. (2008). Simple organic molecules bearing a 3,4-ethylenedioxythiophene linker for efficient dye-sensitized solar cells. Chem. Commun..

