# Supplementary material for: New Oxindole-Bridged Acceptors for Organic Sensitizers: Substitution and Performance Studies in Dye-Sensitized Solar Cells
Source: Molecules. 2020 May 5;25(9):2159. doi: 10.3390/molecules25092159 (PMC7248932; doi:10.3390/molecules25092159)
Supplement: Supplementary file 1 [file molecules-25-02159-s001.pdf]

Supplementary information

New oxindole-bridged acceptors for organic sensitizers: Substitution and performance studies in Dye-Sensitized Solar Cells

Yogesh S. Tingare [1,*], Chaochin Su [1,*], Ming-Tai Shen [2], Sheng-Han Tsai [1], Shih-Yu Ho [1] and Wen-Ren Li [2,*]

[1] Institute of Organic and Polymeric Materials/Research and Development Center for Smart Textile Technology, National Taipei University of Technology, Taipei 10608, Taiwan;
kieferboy8@hotmail.com (S.-H.T.); stacy760414@hotmail.com (S.-Y.H)

[2] Department of Chemistry, National Central University, Chung-Li 32001, Taiwan; moodplayer@hotmail.com

* Correspondence: yogeshtingare@yahoo.co.in (Y.S.T.); f10913@mail.ntut.edu.tw (C.S.);
ch01@ncu.edu.tw (W.-R.L.)

Physicochemical methods.

All organic chemicals were purchased from Sigma–Aldrich, Fluka, Merck, Alfa Aesar, Acros, and Matrix. All solvents were of HPLC grade. NMR spectra were measured using a Bruker 300 or 500 MHz spectrometer with chloroform-*d* and/or dimethyl sulfoxide-d_6 as solvents. UV–Vis spectra were recorded using a Shimadzu UV3600 UV–Vis–NIR spectrophotometer, with CH_2Cl_2 as a solvent. Electrospray ionization mass spectrometry was performed using a JMS-700 HRMS spectrometer. Single-crystal X-ray structural determination was performed using a Bruker KAPPA APEX II apparatus. FTIR spectra were recorded using Perkin Elmer (FTS-40). Cyclic voltammograms were collected using a CHI 627C electrochemical analyzer (CH Instruments).

Electrochemical measurement: Cyclic voltammetry (CV) experiments were performed using an electrochemical setup consisting of a conventional single-compartment three-electrode cell, with a glassy carbon electrode as the working electrode, a silver wire ($Ag/AgNO_3$ in acetonitrile) as the reference electrode, a Pt wire as the counter-electrode, and 0.1 M tetrabutylammonium hexafluorophosphate as the supporting electrolyte. A scan rate of 20 mV s^{-1} was typically used to record the cyclic voltammograms of **TI111-TI116** sensitizers. After each measurement, ferrocene was added as the internal reference for calibration.

Preparation of TiO_2 electrodes and DSSC devices: Two different processes were followed to prepare anatase-TiO_2 (a-TiO_2) sol paste for the dye adsorbed layer and TiO_2 paste for the scattering layer. To prepare a-TiO_2 paste, 14.7 g titanium (IV) *n*-butoxide was mixed with 2 M CH_3COOH (pH = 2.1, 8 mL acetic acid in 64.3 mL distilled water) at room temperature under magnetic stirring for 5 days until a homogeneous sol solution was obtained. The a-TiO_2 sol was then transferred to a 225 mL Teflon-lined autoclave to perform the hydrothermal treatment at 200 ^oC for 5 h. After hydrothermal treatment, the resulting solution was washed and centrifuged with ethanol twice. The precipitate was mixed with 40 mL absolute ethanol and then transferred to a round bottom flask. The resulting solution was then homogenized using a sonicator (Qsonica, Q700) followed by dispersion in the solution of α-terpineol (12.98 g) and ethyl cellulose (10 cps 0.96 g) in anhydrous ethanol (15.04 g). The dispersed solution was concentrated at 50 ^oC under 70 mbar. The a-TiO_2 paste was finalized after grinding by three-roller-miller grinder. The paste preparation for the TiO_2 scattering layer is described as the following: 1.0 g of TiO_2 powder (QF-1125) with particle size around 200~300 nm was dispersed in 40 mL anhydrous ethanol and transferred to a round bottom flask followed by stirring and sonication. α-Terpineol (6.49 g) and the mixture solution of two viscosities of ethyl cellulose (10 cps 0.45 g and 45 cps 0.35 g) in anhydrous ethanol (7.2 g) were added into the above solution, followed by repeated sonication. Finally, the dispersed solution was concentrated by evaporator at 40 ^oC with 70 mbar. The QF-1125 paste was finalized after grinding by three-roller-miller grinder.

The fluorine-doped SnO_2 conduction glass (FTO, TEC7 Hartford glass, transmission ≧ 80%, sheet resistivity: 8 ohm/□, USA) was cleaned with detergent solution, distilled water, acetone, and methanol by ultrasonication each for 30 min, and then dried in oven. To prepare the DSSC working electrodes, a layer of a-TiO_2 paste was coated on FTO by the screen-printing technique, and then dried at 110 °C for 15 min. The coating process was repeated for 4 times to reach the optimal TiO_2 thickness of ~ 13 μm. After screen printing, the electrode was subjected to a programmed sintering process in air as follows: at 125 °C for 15 min, at 325 °C for 5 min, at 375 °C for 5 min, at 450 °C for 15 min, and finally 500 °C for 15 min. The same screen-printing process was repeated twice for coating the QF-1125 TiO_2 paste for the scattering layer. The optimal thickness for the scattering layer was ~ 3 μm. The active working area of the TiO_2 electrodes was 0.16 cm^2. The thickness of TiO_2 adsorption layers and scattering layers were measured by a Force EZstep profiler after each film calcination step.

The TiO_2 working electrode was treated at 80 °C for 15 min before being immersed into various dye solutions (3×10^{-4} M) in a CH_2Cl_2 and kept at 30 °C for 24 h. The Pt counter electrode with mirror finish was prepared by sputtering-deposition (E-1045 ion sputter, Hitachi Ltd., 120 mA current for 30 s) of a 20 nm layer of Pt on top of the FTO substrate. The liquid electrolyte contains 0.1 M lithium iodide (0.2 g), 0.5 M 4-tertbutylpyridine (1.014 g), 0.05 M iodine (0.189 g), and 0.5 M 1, 2-dimethyl-3-propylimidazolium iodide (2.00 g) in 15 mL acetonitrile. To assemble the DSSC device, the dye-sensitized TiO_2 working electrode was placed over the Pt counter electrode, and the edges of the cell were sealed with Dupont Surlyn 1706 (thickness: 60 μm) by heating at 175 °C. The electrolyte was then injected into the intervening space between working and counter electrodes through two holes on the counter electrode, which were covered with a slide glass and sealed with Dupont Surlyn 1706 (thickness : 30 μm).

DSSC performance measurements: The current–voltage characteristics of the DSSC were measured using a Keithley model 2400 source measuring unit. An A-class solar simulator of 300-W Xenon light source (Oriel, #91160) with an AM 1.5 filter (Oriel, #81094) was used. The light intensity at the cell measuring position was adjusted to 100 mW cm^{-2} using an NREL-calibrated monocrystalline silicon solar cell (PVM134 reference cell, PV Measurement, Inc.). The incident photon-to-current conversion efficiencies (IPCEs) were measured by an equipment system, comprised of a 150-W Xenon lamp (Oriel, #66902), a monochromator (Oriel CornerstoneTM 130), and Keithley model 2400 digital source meter.

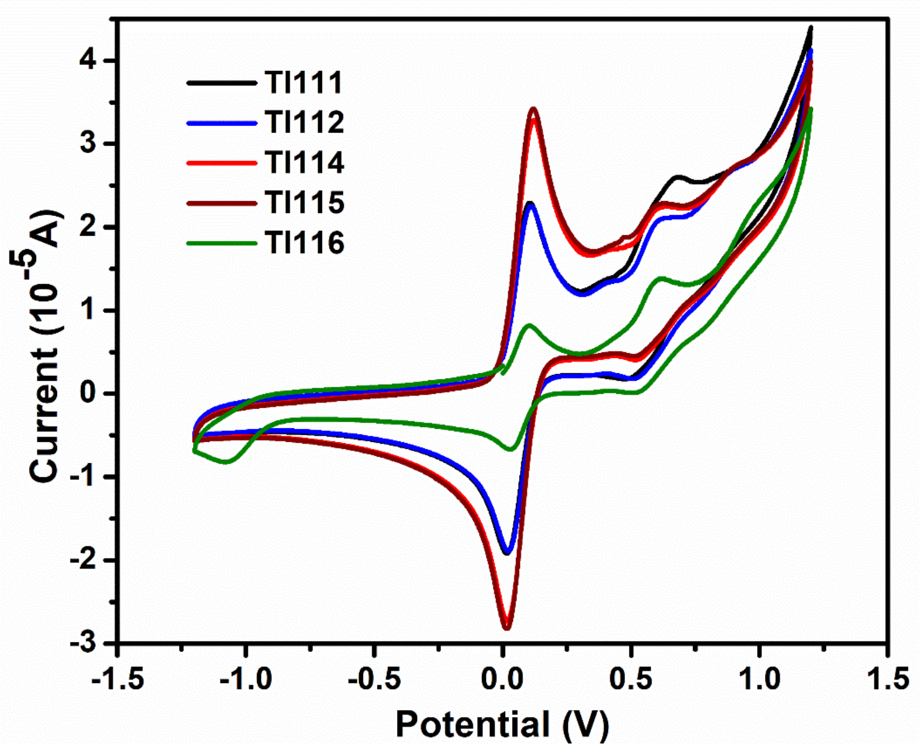

Figure S1: cyclic voltammogram plots for sensitized **TI111-TI116**.

Crystal structure data for compound TI112 ester (4b).

Crystal data for $C_{34}H_{25}FN_2O_3S$ [**4b**]: 0.50 x 0.46 x 0.08 mm^3; *M* = 560.62; monoclinic; space group c1; *a* = 9.6771(4) Å; *b* = 9.7750(4) Å; *c* = 15.3599(6) Å; *α* = 74.2183(13)°; *β* = 83.1520 (16)°; *γ* = 83.2262 (15)°; *V* = 1382.63 (10) Å^3; *Z* = 2; ρ_{calcd} = 1.347 Mg/m^3; *μ* = 0.163 mm^{-1}; F(000) = 584; *T* = 150 (2) K; data collection: 1.38 ≦ θ ≦ 25.00°; reflections collected: 7566; independent reflections: 7566 [R (int) = 0.0000]; refinement method: full-matrix least-squares on F^2; final *R* indices [*I* > 2 (*I*)]: R_1 = 0.0668, wR_2 = 0.1678; *R* indices (all data): R_1 = 0.1117, wR_2 = 0.2083. **CCDC 934260** contains the supplementary crystallographic data for this paper. The data can be obtained free of charge from the Cambridge Crystallographic Data Centre *via* www.ccdc.cam.ac.uk/data_request/cif.

Table S1. Bond lengths [Å] and angles [°] for ester **4b**

**Bond**	**Bond length [Å]**	**Bond**	**Bond length [Å]**
S(1)-C(4)	1.720(3)	C(1)-C(2)	1.373(5)
S(1)-C(1)	1.744(3)	C(1)-C(5)	1.427(5)
F(1)-C(10)	1.366(4)	C(2)-C(3)	1.405(4)
O(1)-C(6)	1.223(4)	C(3)-C(4)	1.376(5)
O(2)-C(33)	1.194(5)	C(4)-C(14)	1.471(5)
O(3)-C(33)	1.316(4)	C(5)-C(7)	1.358(5)
O(3)-C(34)	1.439(5)	C(6)-C(7)	1.488(5)
N(1)-C(20)	1.414(4)	C(7)-C(8)	1.456(4)
N(1)-C(26)	1.422(4)	C(8)-C(13)	1.393(5)
N(1)-C(17)	1.424(4)	C(8)-C(9)	1.394(5)
N(2)-C(6)	1.382(4)	C(9)-C(10)	1.378(5)
N(2)-C(13)	1.407(4)	C(10)-C(11)	1.374(5)
N(2)-C(32)	1.439(4)	C(11)-C(12)	1.386(5)
C(12)-C(13)	1.380(5)	C(26)-N(1)-C(17)	118.9(3)
C(14)-C(19)	1.393(5)	C(6)-N(2)-C(13)	111.0(3)
C(14)-C(15)	1.394(5)	C(6)-N(2)-C(32)	121.7(3)
C(15)-C(16)	1.372(5)	C(13)-N(2)-C(32)	127.3(3)
C(16)-C(17)	1.393(5)	C(2)-C(1)-C(5)	123.3(3)
C(17)-C(18)	1.389(5)	C(2)-C(1)-S(1)	109.3(2)
C(18)-C(19)	1.380(5)	C(5)-C(1)-S(1)	127.4(3)
C(20)-C(21)	1.390(5)	C(1)-C(2)-C(3)	115.1(3)
C(20)-C(25)	1.397(5)	C(4)-C(3)-C(2)	111.6(3)
C(21)-C(22)	1.387(6)	C(3)-C(4)-C(14)	128.3(3)
C(22)-C(23)	1.360(7)	C(3)-C(4)-S(1)	111.9(2)
C(23)-C(24)	1.371(7)	C(14)-C(4)-S(1)	119.8(2)
C(24)-C(25)	1.384(6)	C(7)-C(5)-C(1)	133.7(3)
C(26)-C(31)	1.385(5)	O(1)-C(6)-N(2)	123.9(3)
C(26)-C(27)	1.388(5)	O(1)-C(6)-C(7)	129.6(3)

C(27)-C(28)	1.378(5)	N(2)-C(6)-C(7)	106.5(3)
C(28)-C(29)	1.377(5)	C(5)-C(7)-C(8)	127.3(3)
C(29)-C(30)	1.380(5)	C(5)-C(7)-C(6)	127.1(3)
C(30)-C(31)	1.386(5)	C(8)-C(7)-C(6)	105.6(3)
C(32)-C(33)	1.498(5)	C(13)-C(8)-C(9)	118.9(3)
C(4)-S(1)-C(1)	92.08(16)	C(13)-C(8)-C(7)	108.4(3)
C(33)-O(3)-C(34)	117.6(3)	C(9)-C(8)-C(7)	132.7(3)
C(20)-N(1)-C(26)	120.4(3)	C(10)-C(9)-C(8)	117.3(3)
C(20)-N(1)-C(17)	119.5(3)	F(1)-C(10)-C(11)	118.7(3)
F(1)-C(10)-C(9)	117.4(4)	C(25)-C(20)-N(1)	120.7(3)
C(11)-C(10)-C(9)	123.9(3)	C(22)-C(21)-C(20)	120.5(4)
C(10)-C(11)-C(12)	119.2(3)	C(23)-C(22)-C(21)	120.7(5)
C(13)-C(12)-C(11)	117.8(4)	C(22)-C(23)-C(24)	119.8(4)
C(12)-C(13)-C(8)	123.0(3)	C(23)-C(24)-C(25)	120.8(4)
C(12)-C(13)-N(2)	128.5(3)	C(24)-C(25)-C(20)	120.0(4)
C(8)-C(13)-N(2)	108.6(3)	C(31)-C(26)-C(27)	118.5(3)
C(19)-C(14)-C(15)	116.9(3)	C(31)-C(26)-N(1)	119.8(3)
C(19)-C(14)-C(4)	121.2(3)	C(27)-C(26)-N(1)	121.6(3)
C(15)-C(14)-C(4)	121.9(3)	C(28)-C(27)-C(26)	120.8(4)
C(16)-C(15)-C(14)	122.4(3)	C(29)-C(28)-C(27)	120.8(4)
C(15)-C(16)-C(17)	120.1(3)	C(28)-C(29)-C(30)	118.6(4)
C(18)-C(17)-C(16)	118.3(3)	C(29)-C(30)-C(31)	121.2(4)
C(18)-C(17)-N(1)	120.8(3)	C(26)-C(31)-C(30)	120.0(4)
C(16)-C(17)-N(1)	120.9(3)	N(2)-C(32)-C(33)	113.0(3)
C(19)-C(18)-C(17)	121.0(3)	O(2)-C(33)-O(3)	123.5(4)
C(18)-C(19)-C(14)	121.3(3)	O(2)-C(33)-C(32)	125.6(3)
C(21)-C(20)-C(25)	118.2(3)	O(3)-C(33)-C(32)	110.9(3)
C(21)-C(20)-N(1)	121.1(3)		

Crystal structure data for compound TI116 ester (7).

Crystal data for $C_{34}H_{25}BrN_2O_3S$ [**7**]: 0.30 x 0.10 x 0.04 mm^3; M = 621.53; monoclinic; space group c1; a = 5.2705 (2) Å; b = 15.9713 (6) Å; c = 16.7240 (7) Å; α = 90°; β = 95.670 (2)°; γ = 90°; V = 1400.88 (10) Å^3; Z = 2; ρ_{calcd} = 1.473 Mg/m^3; μ = 1.581 mm^{-1}; F (000) = 636; T = 295 (2) K; data collection: 1.38 ≦ θ ≦ 25.00°; reflections collected: 10789; independent reflections: 4533 [R (int) = 0.0412]; refinement method: full-matrix least-squares on F^2; final R indices [I > 2 (I)]: R_1 = 0.0454, wR_2 = 0.0991; R indices (all data): $R1$ = 0.0591, wR_2 = 0.1077. **CCDC 934272** contains the supplementary crystallographic data for this paper. The data can be obtained free of charge from the Cambridge Crystallographic Data Centre *via* www.ccdc.cam.ac.uk/data_request/cif.

Table S2. Bond lengths [Å] and angles [°] for ester **7**.

Bond	Bond length [Å]	Bond	Bond length [Å]
Br(1)-C(6)	1.906(5)	C(2)-C(3)	1.471(6)
S(1)-C(13)	1.724(5)	C(3)-C(8)	1.376(6)
S(1)-C(10)	1.732(5)	C(3)-C(4)	1.405(7)
O(1)-C(1)	1.235(6)	C(4)-C(5)	1.383(6)
O(2)-C(21)	1.202(6)	C(5)-C(6)	1.372(7)
O(3)-C(21)	1.340(6)	C(6)-C(7)	1.395(8)
O(3)-C(22)	1.437(7)	C(7)-C(8)	1.396(7)
N(1)-C(1)	1.379(6)	C(9)-C(10)	1.440(7)
N(1)-C(4)	1.400(6)	C(10)-C(11)	1.389(7)
N(1)-C(20)	1.447(6)	C(11)-C(12)	1.398(7)
C(1)-C(2)	1.477(7)	C(12)-C(13)	1.369(7)
C(2)-C(9)	1.331(7)	C(13)-C(14)	1.475(7)
C(14)-C(15)	1.354(8)	N(2')-C(29')	1.45(2)
C(14)-C(19)	1.373(8)	C(23')-C(24')	1.380(16)
C(15)-C(16)	1.378(8)	C(23')-C(28')	1.381(19)
C(16)-C(17)	1.374(9)	C(24')-C(25')	1.377(15)
C(17)-C(18)	1.369(9)	C(25')-C(26')	1.408(15)
C(17)-N(2')	1.43(2)	C(26')-C(27')	1.34(2)
C(17)-N(2)	1.46(2)	C(27')-C(28')	1.373(19)
C(18)-C(19)	1.404(8)	C(29')-C(34')	1.37(2)
C(20)-C(21)	1.496(8)	C(29')-C(30')	1.40(2)
N(2)-C(23)	1.42(2)	C(30')-C(31')	1.394(16)
N(2)-C(29)	1.42(2)	C(31')-C(32')	1.47(3)
C(23)-C(24)	1.35(2)	C(32')-C(33')	1.20(2)
C(23)-C(28)	1.43(2)	C(33')-C(34')	1.386(16)
C(24)-C(25)	1.364(15)	C(13)-S(1)-C(10)	92.1(2)
C(25)-C(26)	1.35(2)	C(21)-O(3)-C(22)	116.2(4)
C(26)-C(27)	1.37(2)	C(1)-N(1)-C(4)	110.7(4)
C(27)-C(28)	1.391(16)	C(1)-N(1)-C(20)	121.4(4)
C(29)-C(34)	1.35(2)	C(4)-N(1)-C(20)	127.4(4)
C(29)-C(30)	1.38(2)	O(1)-C(1)-N(1)	122.7(5)
C(30)-C(31)	1.45(3)	O(1)-C(1)-C(2)	129.6(5)
C(31)-C(32)	1.25(3)	N(1)-C(1)-C(2)	107.7(4)
C(32)-C(33)	1.364(15)	C(9)-C(2)-C(3)	124.8(4)
C(33)-C(34)	1.407(16)	C(9)-C(2)-C(1)	130.3(4)
N(2')-C(23')	1.43(2)	C(3)-C(2)-C(1)	104.9(4)
C(8)-C(3)-C(4)	119.8(5)	C(14)-C(15)-C(16)	124.0(6)

C(8)-C(3)-C(2)	132.2(5)	C(17)-C(16)-C(15)	119.8(7)
C(4)-C(3)-C(2)	108.0(4)	C(18)-C(17)-C(16)	118.2(5)
C(5)-C(4)-N(1)	129.2(5)	C(18)-C(17)-N(2')	129.4(7)
C(5)-C(4)-C(3)	122.2(4)	C(16)-C(17)-N(2')	112.4(7)
N(1)-C(4)-C(3)	108.6(4)	C(18)-C(17)-N(2)	110.1(8)
C(6)-C(5)-C(4)	116.5(5)	C(16)-C(17)-N(2)	131.6(8)
C(5)-C(6)-C(7)	123.2(5)	N(2')-C(17)-N(2)	19.3(9)
C(5)-C(6)-Br(1)	118.6(4)	C(17)-C(18)-C(19)	120.2(6)
C(7)-C(6)-Br(1)	118.2(4)	C(14)-C(19)-C(18)	121.8(6)
C(6)-C(7)-C(8)	119.0(5)	N(1)-C(20)-C(21)	112.2(4)
C(3)-C(8)-C(7)	119.3(5)	O(2)-C(21)-O(3)	123.4(5)
C(2)-C(9)-C(10)	134.0(5)	O(2)-C(21)-C(20)	127.5(5)
C(11)-C(10)-C(9)	121.9(4)	O(3)-C(21)-C(20)	109.1(5)
C(11)-C(10)-S(1)	109.7(4)	C(23)-N(2)-C(29)	118.5(19)
C(9)-C(10)-S(1)	128.4(4)	C(23)-N(2)-C(17)	112.1(12)
C(10)-C(11)-C(12)	114.1(5)	C(29)-N(2)-C(17)	129.3(16)
C(13)-C(12)-C(11)	112.5(5)	C(24)-C(23)-N(2)	126.5(17)
C(12)-C(13)-C(14)	127.8(5)	C(24)-C(23)-C(28)	117.4(13)
C(12)-C(13)-S(1)	111.6(4)	N(2)-C(23)-C(28)	116.1(18)
C(14)-C(13)-S(1)	120.5(4)	C(23)-C(24)-C(25)	122.3(12)
C(15)-C(14)-C(19)	115.9(5)	C(26)-C(25)-C(24)	121.6(13)
C(15)-C(14)-C(13)	123.9(5)	C(25)-C(26)-C(27)	118.7(14)
C(19)-C(14)-C(13)	120.1(5)	C(26)-C(27)-C(28)	121.2(13)
C(27)-C(28)-C(23)	118.8(12)	C(28')-C(23')-N(2')	120.1(13)
C(34)-C(29)-C(30)	120.7(14)	C(25')-C(24')-C(23')	120.4(12)
C(34)-C(29)-N(2)	121.0(18)	C(24')-C(25')-C(26')	119.4(11)
C(30)-C(29)-N(2)	118.2(15)	C(27')-C(26')-C(25')	119.5(13)
C(29)-C(30)-C(31)	115.7(18)	C(26')-C(27')-C(28')	120.9(15)
C(32)-C(31)-C(30)	124(3)	C(27')-C(28')-C(23')	120.4(12)
C(31)-C(32)-C(33)	118.5(18)	C(34')-C(29')-C(30')	119.2(14)
C(32)-C(33)-C(34)	122.1(11)	C(34')-C(29')-N(2')	120.4(17)
C(29)-C(34)-C(33)	117.6(14)	C(30')-C(29')-N(2')	120.2(19)
C(17)-N(2')-C(23')	131.8(12)	C(29')-C(30')-C(31')	119.1(13)
C(17)-N(2')-C(29')	113.1(13)	C(30')-C(31')-C(32')	115.7(14)
C(23')-N(2')-C(29')	115.1(18)	C(33')-C(32')-C(31')	124(2)
C(24')-C(23')-C(28')	119.1(12)	C(32')-C(33')-C(34')	121.4(16)
C(24')-C(23')-N(2')	120.8(15)	C(33')-C(34')-C(29')	120.3(13)

Theoretical calculation for the location of HOMOs and LUMOs.

Geometry optimization and the location of the frontier orbitals of **TI111**, **TI112**, **TI114** and **TI116** were computed using the B3LYP/6-31g (d,p) hybrid functional implanted in Gaussian 03. For compound **TI115** B3LYP/LanL2DZ theory level with the GAUSSIAN 03 was used.

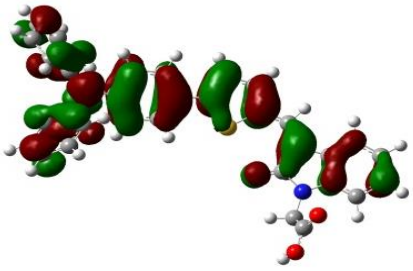 **TI111-HOMO**	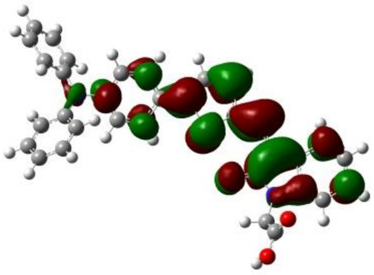 **TI111-LUMO**
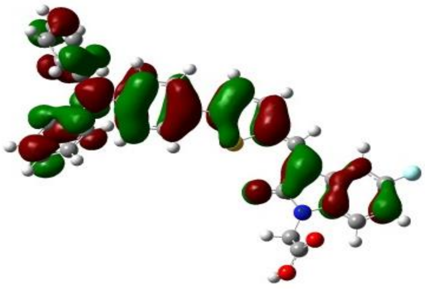 **TI112-HOMO**	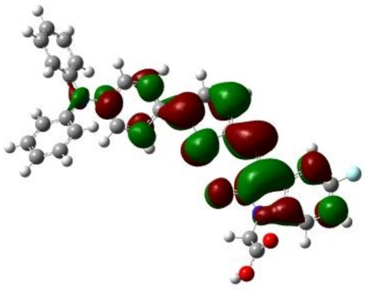 **TI112-LUMO**
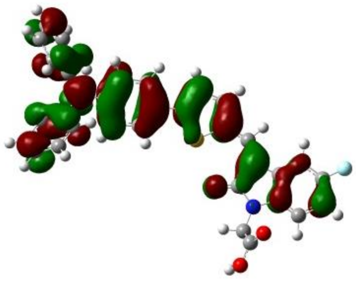 **TI114-HOMO**	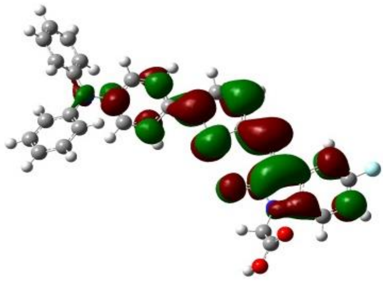 **TI114-LUMO**
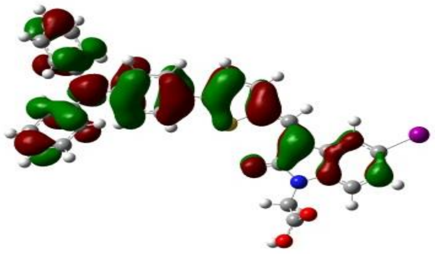 **TI115-HOMO**	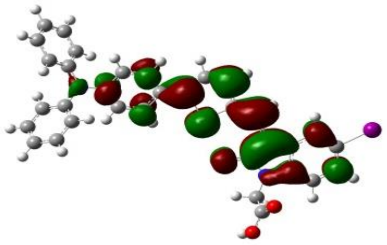 **TI115-LUMO**
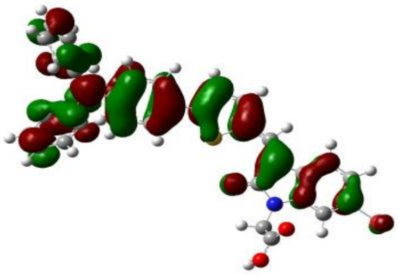 **TI116-HOMO**	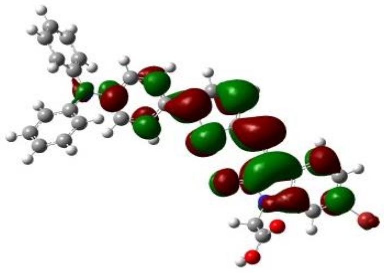 **TI116-LUMO**

Figure S2. Spatial distribution of HOMO-LUMO coefficient of **TI111**, **TI112**, **TI114** and **TI116** at B3LYP/6-31g (d,p) theory level and **TI115** at B3LYP/LanL2DZ theory level with the GAUSSIAN 03.

Table S3. Device optimization of DSSCs based on **TI111-TI116** dyes.

Dye	Thikness	J_{sc}	V_{oc}	*FF*	η%
**TI111**	12+3	8.24	700	0.692	4.23
**TI112**		11.40	710	0.698	5.57
**TI114**		10.63	690	0.710	5.65
**TI115**		10.49	680	0.721	5.37
**TI116**		9.24	650	0.685	4.31
**TI111**	**13+3**	**10.03**	**680**	**0.669**	**4.76**
**TI112**		**11.32**	**690**	**0.695**	**5.43**
**TI114**		**12.46**	**720**	**0.708**	**6.35**
**TI115**		**11.97**	**680**	**0.714**	**5.81**
**TI116**		**9.66**	**630**	**0.692**	**4.21**
**TI111**	14+3	10.05	710	0.720	4.93
**TI112**		10.48	690	0.692	5.00
**TI114**		12.30	710	0.719	6.28
**TI115**		10.57	660	0.706	5.23
**TI116**		8.08	610	0.691	3.63

Table S4. EIS values for DSSCs based on **TI111-TI116** dyes.

**Dyes**	R_k	R_w	L_{um}	k_{eff}	$\tau(s^{-1})$	R_k/R_w	L_n (um)	D_{eff} (cm^2/s)	η%
**TI111**	12.2	8.6	16	9.479	0.10549636	1.41860465	19.056830	3.44242E-05	4.76
**TI112**	11	5.8	16	8.108	0.12332433	1.89655172	22.034455	3.93691E-05	5.43
**TI114**	9.5	4.85	16	8.108	0.12332433	1.95876288	22.392929	4.06605E-05	6.35
**TI115**	10.1	5.2	16	9.479	0.10549636	1.94230769	22.298671	4.71325E-05	5.81
**TI116**	13.2	6.1	16	17.12	0.05841121	2.16393442	23.536508	9.48392E-05	4.21

^{1}H and ^{13}C NMR spectra of TI111-TI116 sensitizers.

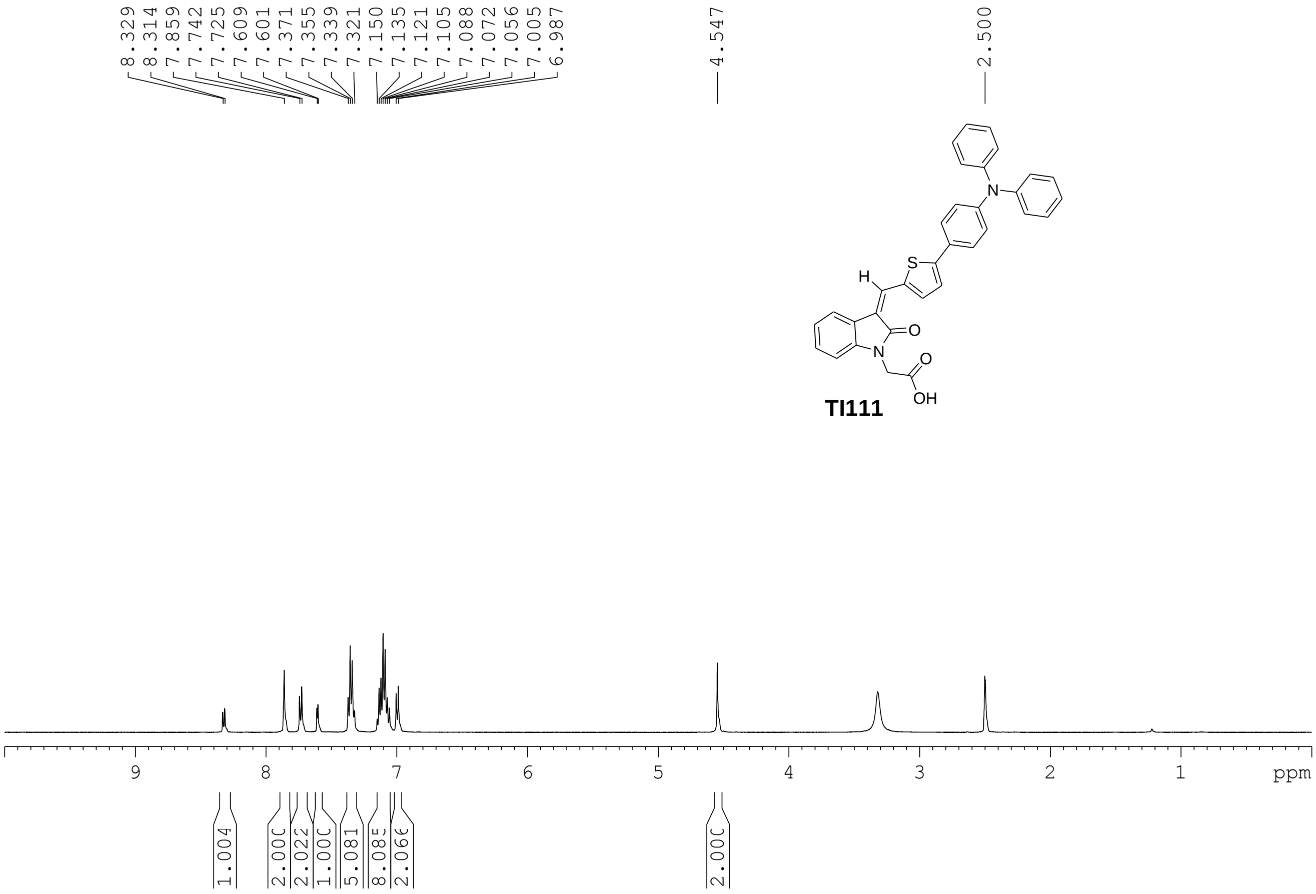

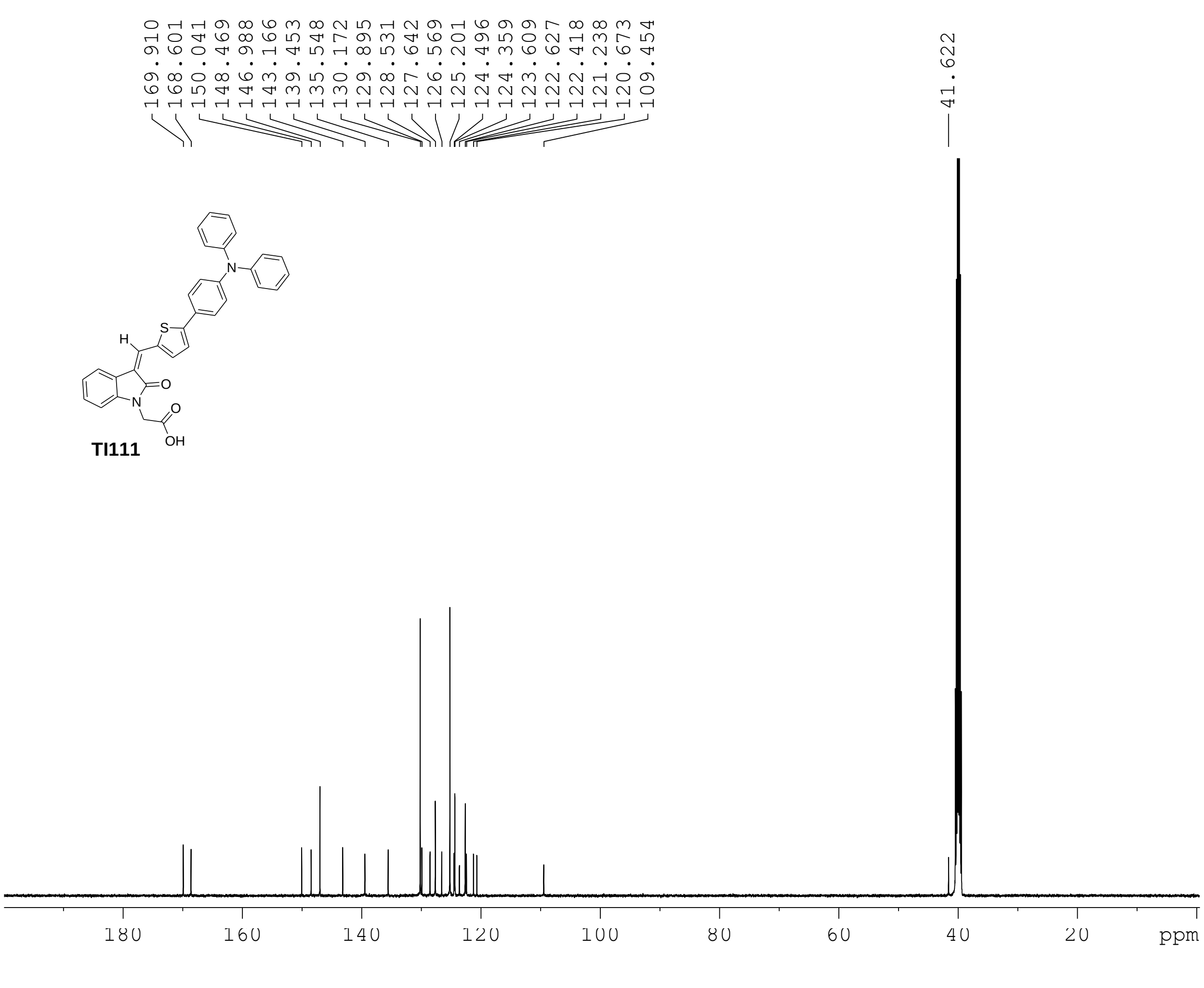

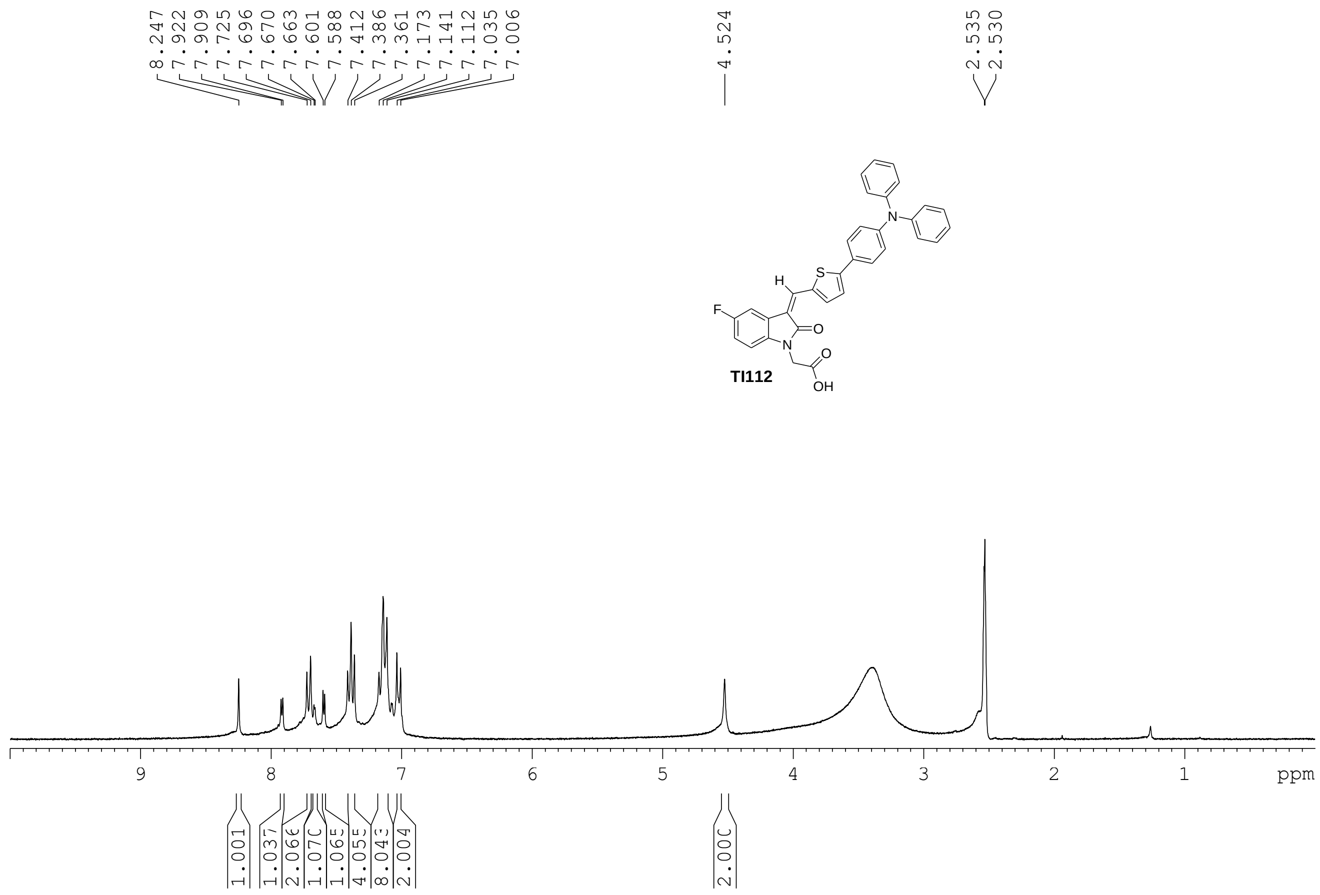
8.247
7.922
7.909
7.725
7.696
7.670
7.663
7.601
7.588
7.412
7.386
7.361
7.173
7.141
7.112
7.035
7.006
4.524
2.535
2.530
TI112
ppm

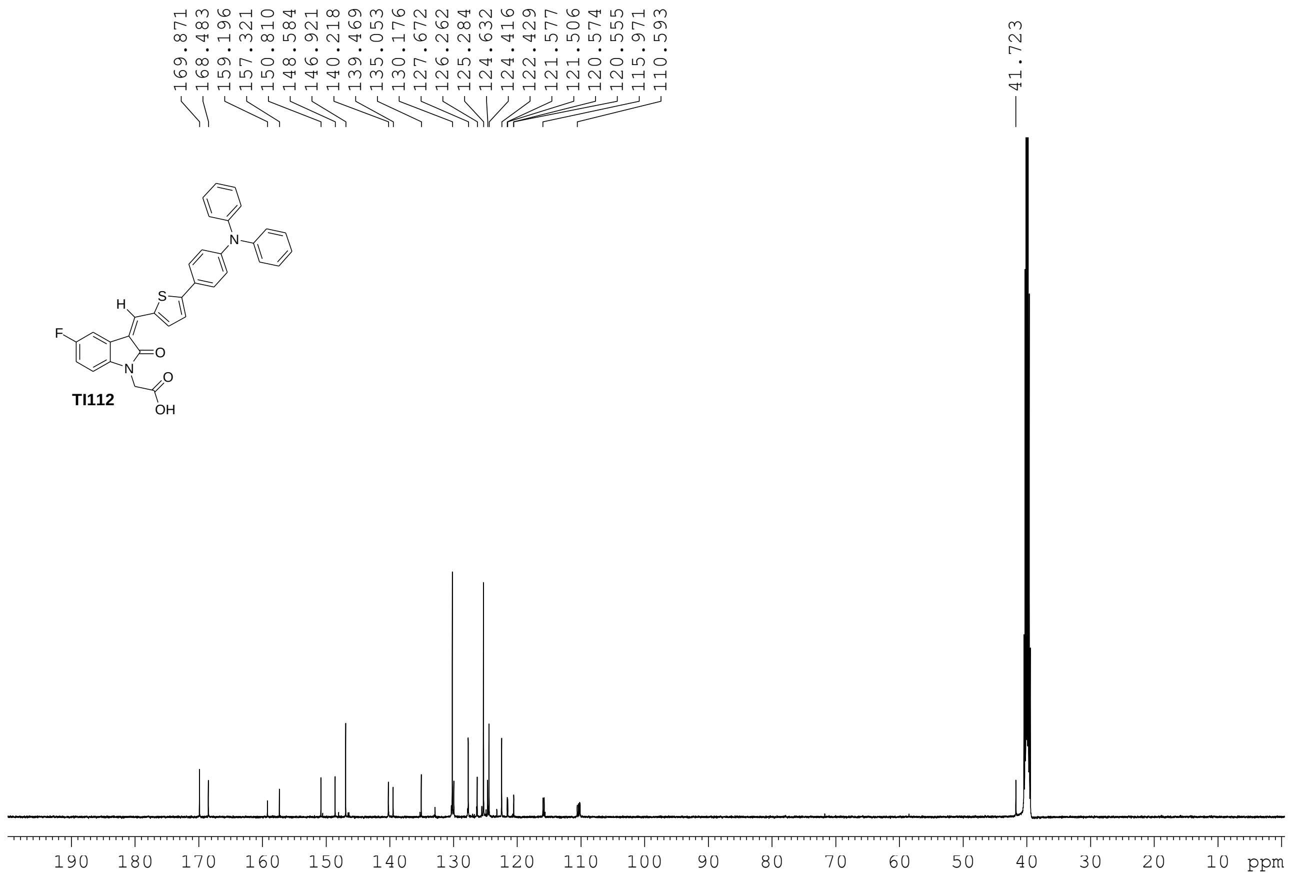
169.871
168.483
159.196
157.321
150.810
148.584
146.921
140.218
139.469
135.053
130.176
127.672
126.262
125.284
124.632
124.416
122.429
121.577
121.506
120.574
120.555
115.971
110.593
41.723
TI112
ppm

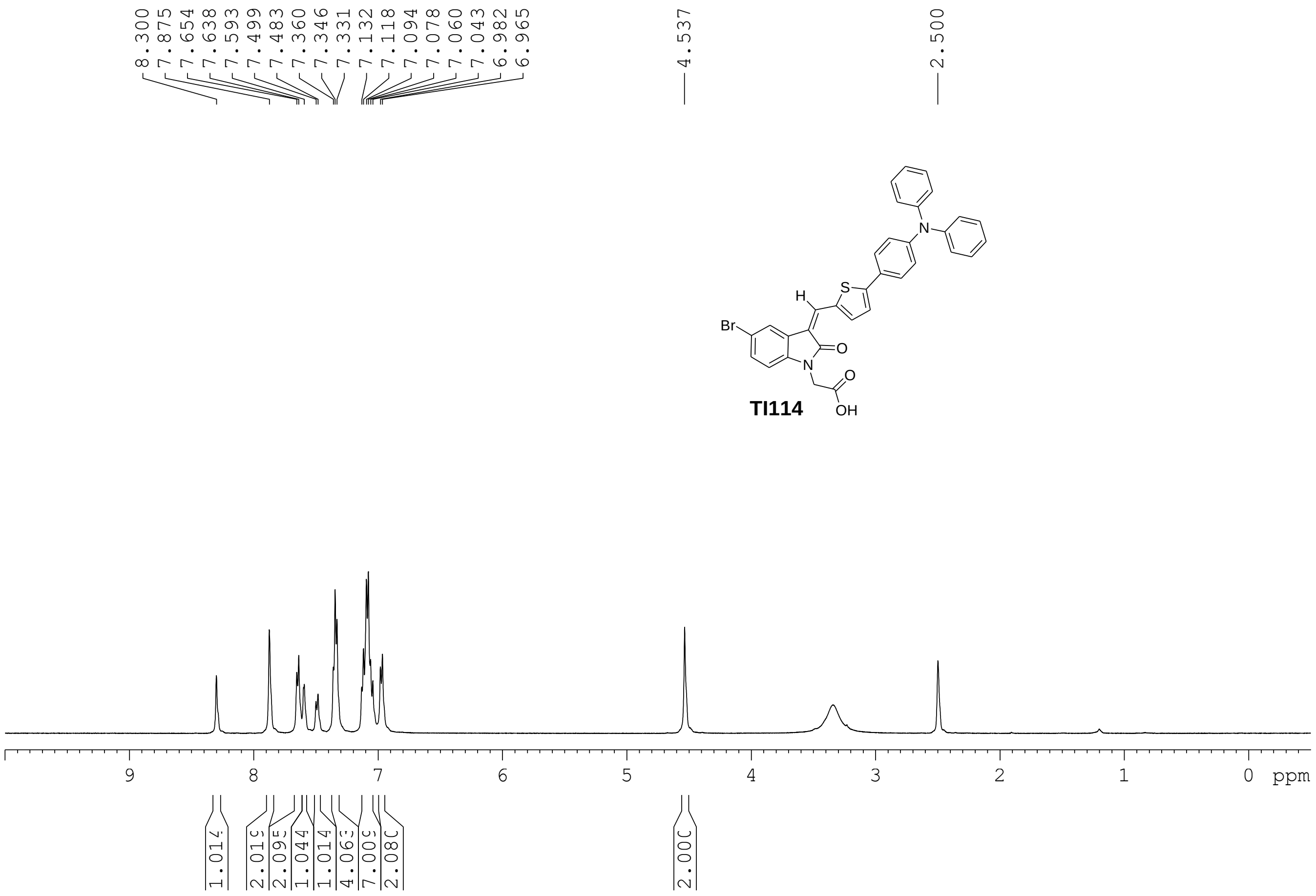
8.300
7.875
7.654
7.638
7.593
7.499
7.483
7.360
7.346
7.331
7.132
7.118
7.094
7.078
7.060
7.043
6.982
6.965
4.537
2.500
Br
H
S
N
O
OH
TI114
9
8
7
6
5
4
3
2
1
0 ppm

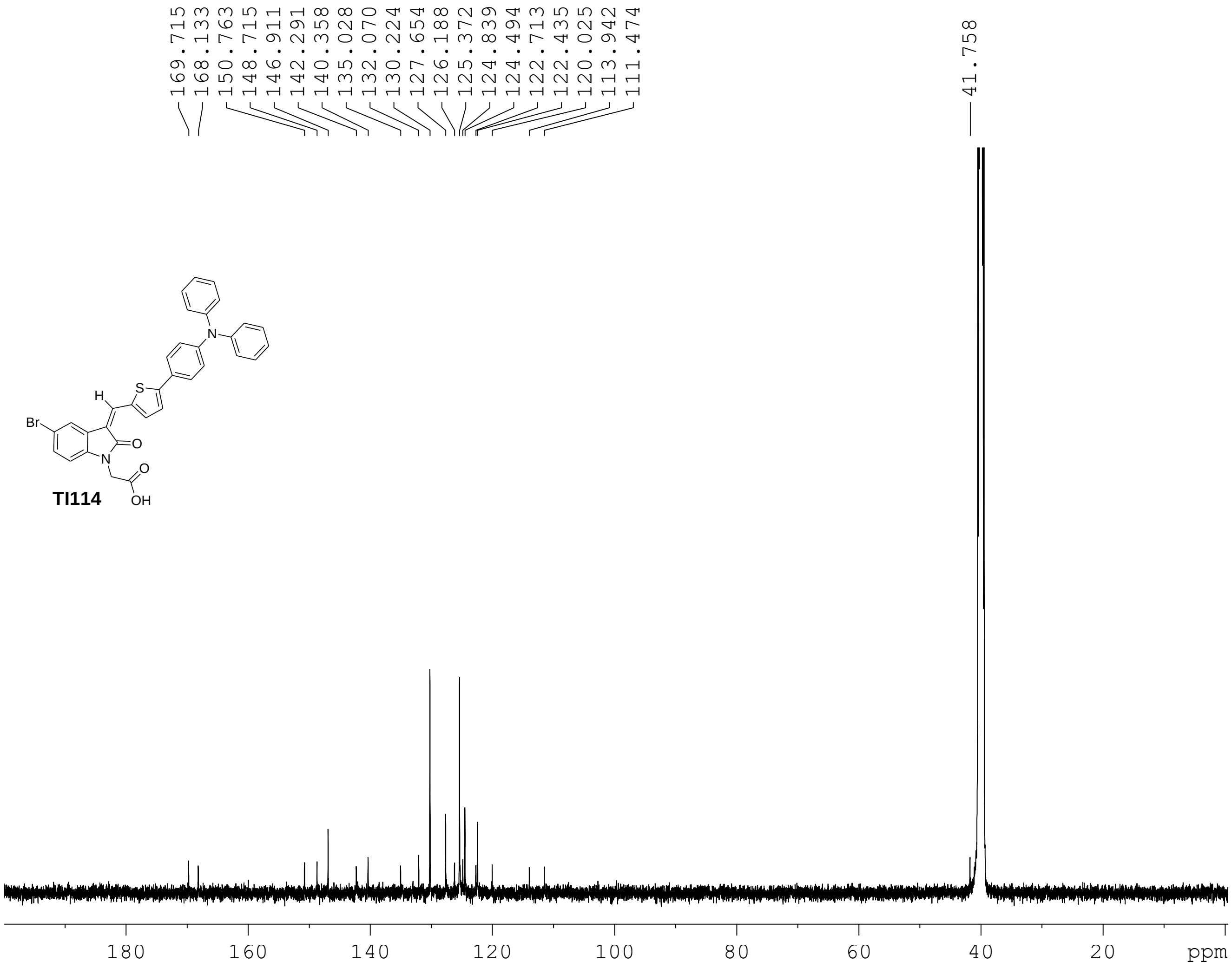
169.715
168.133
150.763
148.715
146.911
142.291
140.358
135.028
132.070
130.224
127.654
126.188
125.372
124.839
124.494
122.713
122.435
120.025
113.942
111.474
41.758
Br
H
S
N
O
OH
TI114
180
160
140
120
100
80
60
40
20
ppm

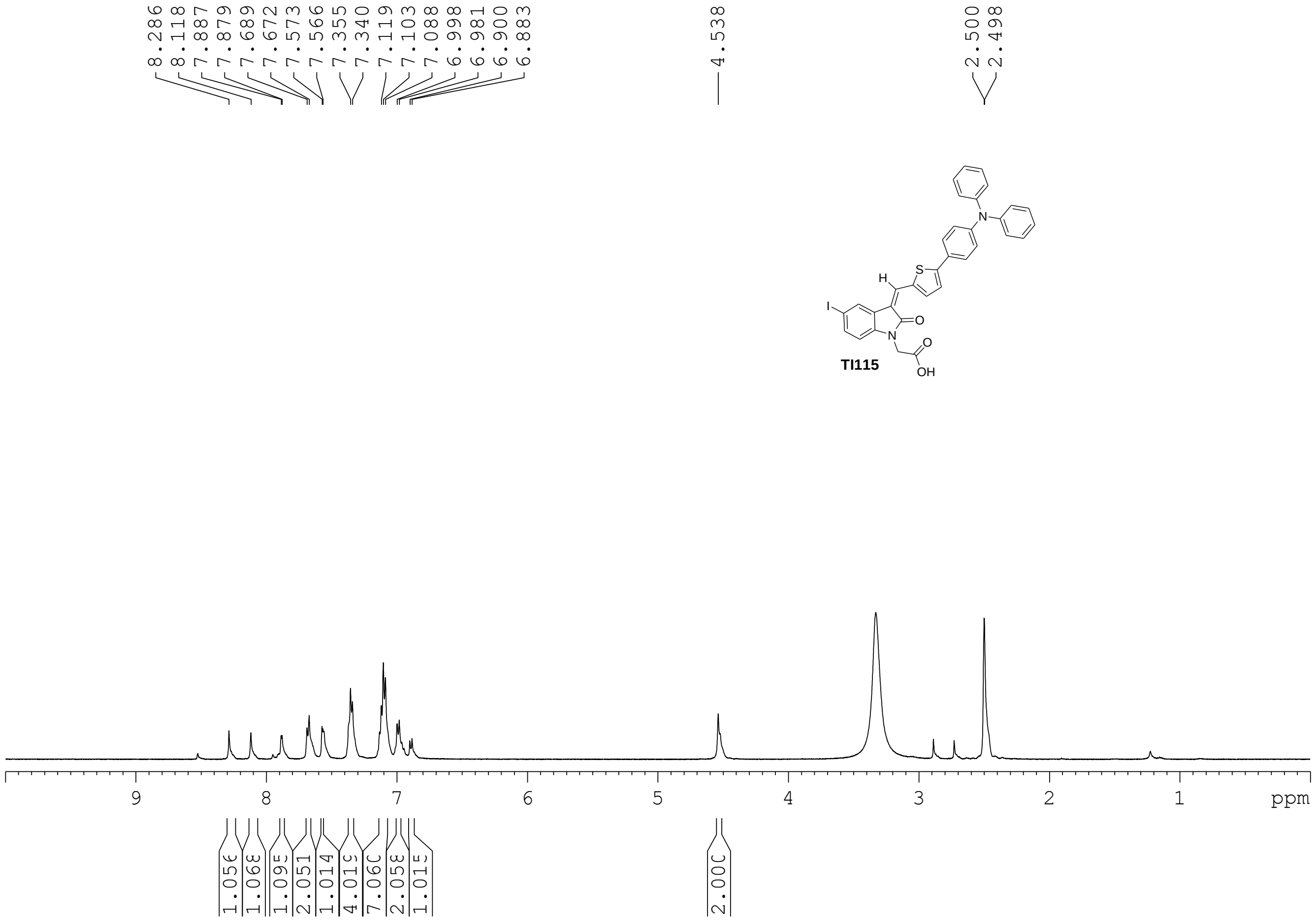
8.286
8.118
7.887
7.879
7.689
7.672
7.573
7.566
7.355
7.340
7.119
7.103
7.088
6.998
6.981
6.900
6.883
4.538
2.500
2.498
I
H
S
N
O
OH
TI115
9 8 7 6 5 4 3 2 1 ppm
2.051
1.014
2.000

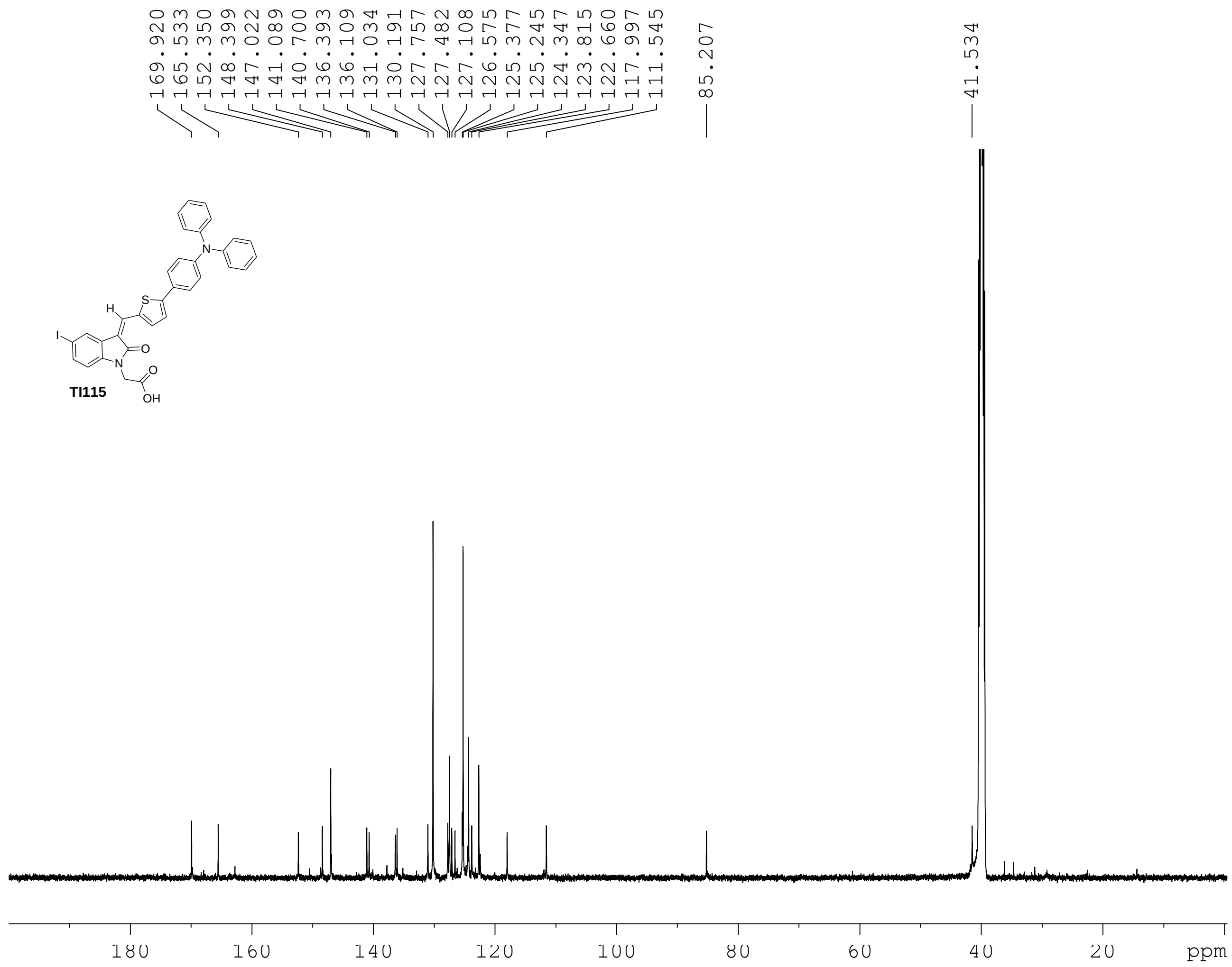
169.920
165.533
152.350
148.399
147.022
141.089
140.700
136.393
136.109
131.034
130.191
127.757
127.482
127.108
126.575
125.377
125.245
124.347
123.815
122.660
117.997
111.545
85.207
41.534
I
H
S
N
O
OH
TI115
180 160 140 120 100 80 60 40 20 ppm

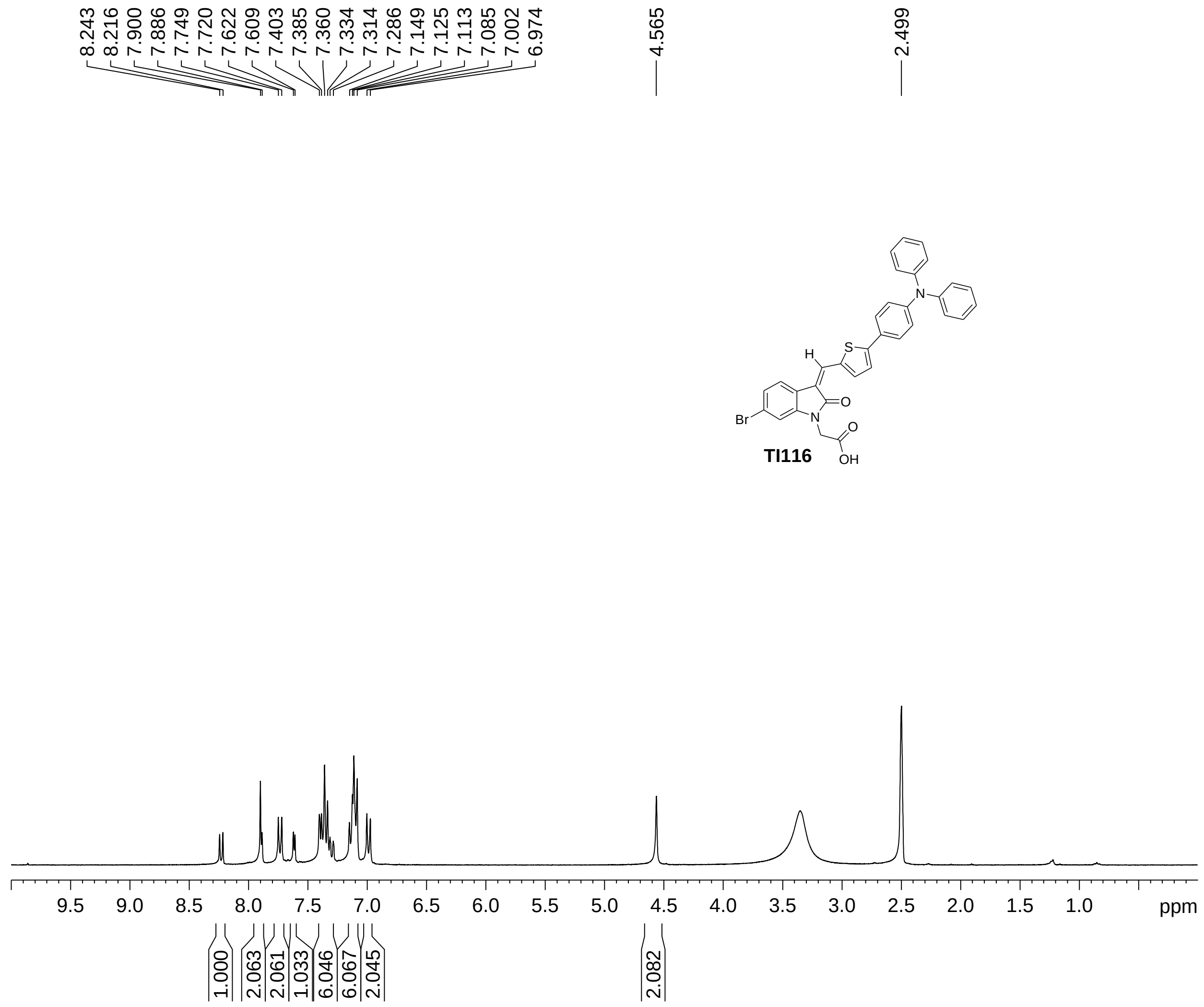
8.243
8.216
7.900
7.886
7.749
7.720
7.622
7.609
7.403
7.385
7.360
7.334
7.314
7.286
7.149
7.125
7.113
7.085
7.002
6.974
4.565
2.499
TI116
Br
N
O
OH
H
S
9.5 9.0 8.5 8.0 7.5 7.0 6.5 6.0 5.5 5.0 4.5 4.0 3.5 3.0 2.5 2.0 1.5 1.0 ppm
1.000
2.063
2.061
1.033
6.046
6.067
2.045
2.082

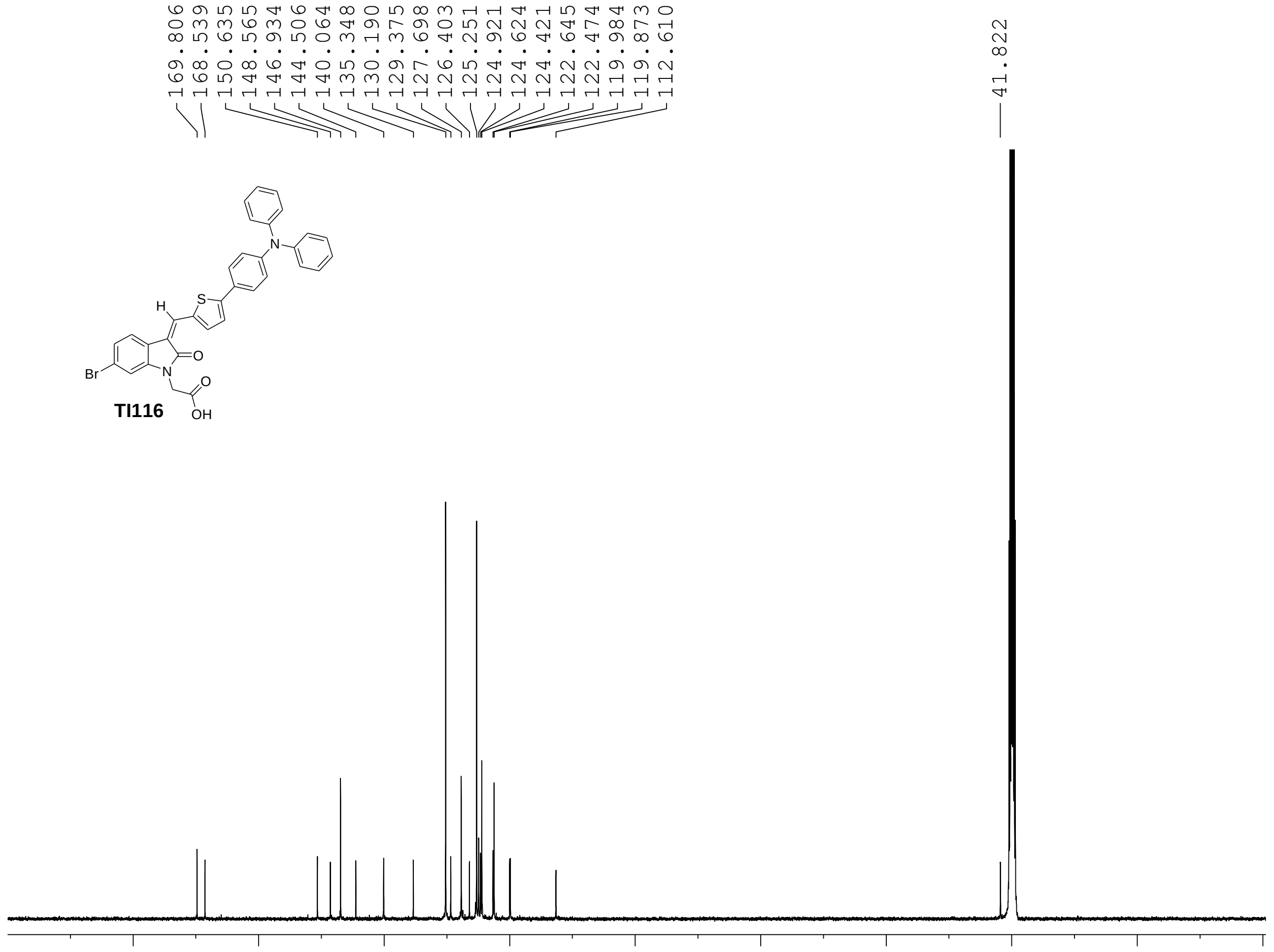
169.806
168.539
150.635
148.565
146.934
144.506
140.064
135.348
130.190
129.375
127.698
126.403
125.251
124.921
124.624
124.421
122.645
122.474
119.984
119.873
112.610
41.822
TI116
Br
N
O
OH
H
S
180 160 140 120 100 80 60 40 20 ppm
